# Supplementary material for: A systematic review and meta-analysis of the associations between interparental and sibling relationships: Positive or negative?
Source: PLoS One. 2021 Sep 28;16(9):e0257874. doi: 10.1371/journal.pone.0257874 (PMC8478168; doi:10.1371/journal.pone.0257874)
Supplement: S1 Table — (DOCX) [file pone.0257874.s002.docx]

**S1 Table**

*Key search terms for literature research*

| Parent terms | Sibling terms | Relationship quality terms (positive dimensions) | Relationship quality terms (negative dimensions) |
| --- | --- | --- | --- |
| Parent*, couple*, partner*, dyadic, intimate, marital | Sibling*, brother*, sisters* | Relation*, relationship quality/satisfaction/adjustment/functioning, communication quality, interaction quality, cohesion, warmth, intimacy, positivity | Conflict, distress, discord, disagreements, coercion, tension, hostility, aggression, violence, bullying, abuse, negativity |

**Example full electronic search query: Web of Science**

TS=((parent* OR couple OR partner OR intimate OR marital OR dyadic) NEAR/5 (relation* OR "relationship quality" OR "relationship satisfaction" OR "relationship adjustment" OR "relationship functioning" OR "communication quality" OR interaction OR quality OR cohesion OR warmth OR intimacy OR positivity OR conflict OR distress OR discord OR disagreement OR hostility OR aggression OR violence OR bullying OR coercion OR abuse OR negativity OR tension)) AND TS=((sibling OR brother OR sister) NEAR/5 (relation* OR "relationship quality" OR "relationship satisfaction" OR "relationship adjustment" OR "relationship functioning" OR "communication quality" OR interaction OR quality OR cohesion OR warmth OR intimacy OR positivity OR conflict OR distress OR discord OR disagreement OR hostility OR aggression OR violence OR bullying OR coercion OR abuse OR negativity OR tension))
