# Supplementary material for: A systematic review and meta-analysis of the associations between interparental and sibling relationships: Positive or negative?
Source: PLoS One. 2021 Sep 28;16(9):e0257874. doi: 10.1371/journal.pone.0257874 (PMC8478168; doi:10.1371/journal.pone.0257874)
Supplement: S2 Table — (DOCX) [file pone.0257874.s003.docx]

**S2 Table**

*Study characteristics of included studies (k = 47)*

| **Authors** | **Publication year** | **Country of study** | **Sample^a^** | **Interparental relationship measure  (*M; SD*)^b^** | **Sibling relationship measure  (*M; SD*)^b^** | **Interparental-sibling association** |
| --- | --- | --- | --- | --- | --- | --- |
| Beyers Carlson | 2018 | USA | *N* = 106 families | Braiker & Kelley Intimate Relationship Scale BKIRS, positive relationship quality subscale (6.60; .81) | Observer rating of sibling sharing (0; 2.45) | *r* = -.20 |
|  |  |  |  | Braiker & Kelley Intimate Relationship Scale BKIRS, negative relationship quality subscale (3.00; .92) | Observer rating of sibling sharing (0; 2.45) | *r* = .05^c^ |
| Brockman | 1994 | USA | *N* = 91 families | Dyadic Adjustment Scale DAS | Sibling Inventory of Differential Experience SIDE | *r* = .00^d^ |
| Brody et al. | 1987 | USA | *N* = 42 mothers and their same sex children | Dyadic Adjustment Scale DAS  (116.63; 12.97) | Observer rating of prosocial behavior  (26.51; 19.72) | *r* = .22 |
|  |  |  |  |  | Observer rating of agonistic behavior  (25.72; 16.84) | *r* = .44^c^ |
|  |  |  |  | O'Leary-Porter Scale OPS (31.37; 4.34) | Observer rating of prosocial behavior  (26.51; 19.72) | *r* = .33^c^ |
|  |  |  |  |  | Observer rating of agonistic behavior  (25.72; 16.84) | *r* = .33 |
| Brody et al. | 1994 | USA | *N* = 71 same sex dyads and their parents | Dyadic Adjustment Scale DAS at Time 1 | SRQ Sibling Relationship Questionnaire, positivity subscale, at Time 2 | *r* = .35 |
|  |  |  |  |  | SRQ Sibling Relationship Questionnaire, negativity subscale, at Time 2 | *r* = .38^c^ |
|  |  |  |  |  | SRQ Sibling Relationship Questionnaire, positivity subscale, at Time 3 | *r* = .20 |
|  |  |  |  |  | SRQ Sibling Relationship Questionnaire, negativity subscale, at Time 3 | *r* = .09^c^ |
|  |  |  |  | O'Leary-Porter Scale OPS at Time 1 | SRQ Sibling Relationship Questionnaire, positivity subscale, at Time 2 | *r* = -.29^c^ |
|  |  |  |  |  | SRQ Sibling Relationship Questionnaire, negativity subscale, at Time 2 | *r* = -.40 |
|  |  |  |  |  | SRQ Sibling Relationship Questionnaire, positivity subscale, at Time 3 | *r* = -.29 |
|  |  |  |  |  | SRQ Sibling Relationship Questionnaire, negativity subscale, at Time 3 | *r* = -.18 |

**Table S2 continued**

| **Authors** | **Publication year** | **Country of study** | **Sample^a^** | **Interparental relationship measure  (M; SD)^b^** | **Sibling relationship measure  (M; SD)^b^** | **Interparental-sibling association** |
| --- | --- | --- | --- | --- | --- | --- |
| Button & Gealt | 2010 | USA | *N* = 8122 students | Unpublished measure of witnessing domestic violence | Unpublished measure of sibling violence | OR = 2.064 |
| Caya | 2001 | USA | *N* = 89 students | O'Leary-Porter Scale OPS (10.12; 6.37) | Network of Relationships Inventory NRI, sibling support subscale (22.14; 6.57) | *r* = .019^c^ |
| Conners | 1999 | USA | *N* = 70 adolescent girls | Parenting Alliance Measure PAM  (81.2; 13.1) | Sibling Relationship Questionnaire SRQ, mother reported warmth (3.31; .62) | *r* = .197 |
|  |  |  |  |  | Sibling Relationship Questionnaire SRQ, mother reported conflict (2.78; .77) | *r* = .311^c^ |
|  |  |  |  |  | Sibling Relationship Questionnaire SRQ, daughter reporter warmth (3.26; .72) | *r* = .042 |
|  |  |  |  |  | Sibling Relationship Questionnaire SRQ, daughter reported conflict (3.00; .70) | *r* = .149^c^ |
|  |  |  |  | Revised Dyadic Adjustment Scale RDAS (56.4; 8.0) | Sibling Relationship Questionnaire SRQ, mother reported warmth (3.31; .62) | *r* = -.022 |
|  |  |  |  |  | Sibling Relationship Questionnaire SRQ, mother reported conflict (2.78; .77) | *r* = .397^c^ |
|  |  |  |  |  | Sibling Relationship Questionnaire SRQ, daughter reporter warmth (3.26; .72) | *r* = .037 |
|  |  |  |  |  | Sibling Relationship Questionnaire SRQ, daughter reported conflict (3.00; .70) | *r* = .046^c^ |
| Dantchev & Wolke | 2019 | UK | *N* = 6838 children | Unpublished measure of conflicting partnership (2.24; 1.88) | Categorial measure of sibling bullying involvement (28.1% involved) | *r* = .072 |
|  |  |  |  | Categorial measure of domestic violence (25.4% present) |  | OR = 1.33 |
| Davies et al. | 2018 | USA | *N* = 236 families | Observer rating of interparental conflict (9.60; 3.62) | Sibling Interview for Mothers SIM  (8.63; 3.00) | *r* = .07^c^ |
| Dawson et al. | 2014 | UK | *N* = 124 families | Golombok-Rust Inventory of Marital State GRIMS, mother rated (24.83; 10.54) | Berkley Puppet Interview BPI (4.23; 1.12) | *r* = .22 |
|  |  |  |  | Golombok-Rust Inventory of Marital State GRIMS, father rated (24.01; 9.74) |  | *r* = .24 |

**Table S2 continued**

| **Authors** | **Publication year** | **Country of study** | **Sample^a^** | **Interparental relationship measure  (M; SD)^b^** | **Sibling relationship measure  (M; SD)^b^** | **Interparental-sibling association** |
| --- | --- | --- | --- | --- | --- | --- |
| Dekovic & Buist | 2005 | Netherlands | *N* = 288 families | Modified version of the Inventory of Parent and Peer Attachment IPPA | Modified version of the Inventory of Parent and Peer Attachment IPPA | *r* = .10 |
| Dunn et al. | 1999 | UK | *N* = 3681 sibling pairs | Unpublished measure of affection rated by partner at Time 1 | Maternal Interview by Stocker et al. (1989), positivity subscale at Time 2 | *r* = .11 |
|  |  |  |  |  | Maternal Interview by Stocker et al. (1989), negativity subscale at Time 2 | *r* = .12^c^ |
|  |  |  |  | Unpublished measure of affection rated by mother at Time 1 | Maternal Interview by Stocker et al. (1989), positivity subscale at Time 2 | *r* = .11 |
|  |  |  |  |  | Maternal Interview by Stocker et al. (1989), negativity subscale at Time 2 | *r* = .13^c^ |
|  |  |  |  | Unpublished measure of hostility rated by partner at Time 1 | Maternal Interview by Stocker et al. (1989), positivity subscale at Time 2 | *r* = .01^c^ |
|  |  |  |  |  | Maternal Interview by Stocker et al. (1989), negativity subscale at Time 2 | *r* = .13 |
|  |  |  |  | Unpublished measure of hostility rated by mother at Time 1 | Maternal Interview by Stocker et al. (1989), positivity subscale at Time 2 | *r* = .05^c^ |
|  |  |  |  |  | Maternal Interview by Stocker et al. (1989), negativity subscale at Time 2 | *r* = .19 |
| Erel et al. | 1998 | USA | *N* = 73 sibling pairs | Aggregation of the Conflict Tactics Scale CTS and the O'Leary Porter Scale OPS  (.00; 2.59) | Observer rating of negative sibling interaction (.94; .77) | *r* = .15 |
|  |  |  |  |  | Observer rating of positive sibling interaction (1.64; .95) | *r* = -.06^c^ |
|  |  |  |  | Aggregation of the Dyadic Adjustment Scale DAS and the Family Adaptability and Cohesion Scale FACES III (0.00; .97) |  | *r* = .06^c^ |
|  |  |  |  |  |  | *r* = .14 |
| Grych et al. | 2004 | USA | *N* = 388 adolescents | Children’s Perception of Interparental Conflict Questionnaire CPIC, conflict properties subscale (18.11; 9.90) | Brother Sister Questionnaire, empathy and similarity subscales (70.09; 12.21) | *r* = .16^c^ |

**Table S2 continued**

| **Authors** | **Publication year** | **Country of study** | **Sample^a^** | **Interparental relationship measure  (M; SD)^b^** | **Sibling relationship measure  (M; SD)^b^** | **Interparental-sibling association** |
| --- | --- | --- | --- | --- | --- | --- |
| Guinn et al. | 2012 | USA | *N* = 75 parents and *N* = 41 boys | Dyadic Adjustment Scale DAS (50.13; 7.97) | Sibling Relationship Questionnaire SRQ, warmth subscale (2.76; .75) | *r* = .09 |
|  |  |  | *N* = 75 parents and  *N* = 34 girls | Dyadic Adjustment Scale DAS (50.13; 7.97) | Sibling Relationship Questionnaire SRQ, warmth subscale (3.27; .80) | *r* = .25 |
|  |  |  | *N* = 75 parents and *N* = 41 boys | Dyadic Adjustment Scale DAS (50.13; 7.97) | Sibling Relationship Questionnaire SRQ, conflict subscale (3.21; 1.08) | *r* = -.17^c^ |
|  |  |  | *N* = 75 parents and  *N* = 34 girls | Dyadic Adjustment Scale DAS (50.13; 7.97) | Sibling Relationship Questionnaire SRQ, conflict subscale (2.58; .98) | *r* = .28^c^ |
|  |  |  | *N* = 75 parents and *N* = 41 boys | Dyadic Adjustment Scale DAS (50.13; 8.29) | Sibling Relationship Questionnaire SRQ, warmth subscale (2.76; .75) | *r* = .14 |
|  |  |  | *N* = 75 parents and  *N* = 34 girls | Dyadic Adjustment Scale DAS (50.13; 8.29) | Sibling Relationship Questionnaire SRQ, warmth subscale (3.27; .80) | *r* = -.03 |
|  |  |  | *N* = 75 parents and *N* = 41 boys | Dyadic Adjustment Scale DAS (50.13; 8.29) | Sibling Relationship Questionnaire SRQ, conflict subscale (3.21; 1.08) | *r* = -.04^c^ |
|  |  |  | *N* = 75 parents and  *N* = 34 girls | Dyadic Adjustment Scale DAS (50.13; 8.29) | Sibling Relationship Questionnaire SRQ, conflict subscale (2.58; .98) | *r* = .09^c^ |
| Haj-Yahia & Abdo-Kaloti | 2003 | Israel | *N* = 1185 adolescents | Conflict Tactics Scale CTS, psychological aggression subscale at time 1, father-to-mother | Conflict Tactics Scale CTS, psychological aggression subscale at time 1 | *r* = .63 |
|  |  |  |  |  | CTS, physical violence subscale at time 1 | *r* = .54 |
|  |  |  |  |  | CTS, psychological aggression subscale at time 2 | *r* = .59 |
|  |  |  |  |  | CTS, physical violence subscale at time 2 | *r* = .44 |
|  |  |  |  | CTS, physical violence subscale at time 1, father-to-mother | CTS, psychological aggression subscale at time 1 | *r* = .45 |
|  |  |  |  |  | CTS, physical violence subscale at time 1 | *r* = .46 |
|  |  |  |  |  | CTS, psychological aggression subscale at time 2 | *r* = .37 |
|  |  |  |  |  | CTS, physical violence subscale at time 2 | *r* = .36 |
|  |  |  |  | CTS, psychological aggression subscale at time 2, father-to-mother | CTS, psychological aggression subscale at time 2 | *r* = .52 |
|  |  |  |  |  | CTS, physical violence subscale at time 2 | *r* = .40 |

**Table S2 continued**

| **Authors** | **Publication year** | **Country of study** | **Sample^a^** | **Interparental relationship measure  (M; SD)^b^** | **Sibling relationship measure  (M; SD)^b^** | **Interparental-sibling association** |
| --- | --- | --- | --- | --- | --- | --- |
|  |  |  |  | CTS, physical violence subscale at time 2, father-to-mother | CTS, psychological aggression subscale at time 2 | *r* = .29 |
|  |  |  |  |  | CTS, physical violence subscale at time 2 | *r* = .31 |
|  |  |  |  | CTS, psychological aggression subscale at time 1, mother-to-father | CTS, psychological aggression subscale at time 1 | *r* = .54 |
|  |  |  |  |  | CTS, physical violence subscale at time 1 | *r* = .50 |
|  |  |  |  |  | CTS, psychological aggression subscale at time 2 | *r* = .51 |
|  |  |  |  |  | CTS, physical violence subscale at time 2 | *r* = .41 |
|  |  |  |  | CTS, physical violence subscale at time 1, mother-to-father | CTS, psychological aggression subscale at time 1 | *r* = .17 |
|  |  |  |  |  | CTS, physical violence subscale at time 1 | *r* = .31 |
|  |  |  |  |  | CTS, psychological aggression subscale at time 2 | *r* = .13 |
|  |  |  |  |  | CTS, physical violence subscale at time 2 | *r* = .28 |
|  |  |  |  | CTS, psychological aggression subscale at time 2, mother-to-father | CTS, psychological aggression subscale at time 2 | *r* = .48 |
|  |  |  |  |  | CTS, physical violence subscale at time 2 | *r* = .38 |
|  |  |  |  | CTS, physical violence subscale at time 2, mother-to-father | CTS, psychological aggression subscale at time 2 | *r* = .12 |
|  |  |  |  |  | CTS, physical violence subscale at time 2 | *r* = .25 |
| Hakvoort et al. | 2010 | Netherlands | *N* = 88 families | Combined Marital Satisfaction Scale MSS & Parental Stress Index PSI, Relationship with Spouse subscale (4.86; 1.13) | Sibling Relationship Inventory SRI & Leiden Sibling Relationship Questionnaire LRSQ, affection subscales (2.28; .34) | *r* = .14 |
|  |  |  |  |  | Sibling Relationship Inventory SRI & Leiden Sibling Relationship Questionnaire LRSQ, quarrelling subscales (1.79; .39) | *r* = .20^c^ |

**Table S2 continued**

| **Authors** | **Publication year** | **Country of study** | **Sample^a^** | **Interparental relationship measure  (M; SD)^b^** | **Sibling relationship measure  (M; SD)^b^** | **Interparental-sibling association** |
| --- | --- | --- | --- | --- | --- | --- |
| Hindman et al. | 2013 | USA | *N* = 86 families | Dyadic Adjustment Scale DAS, father-rated (114.27; 14.36) | Sibling Relationship Questionnaire SRQ, intimacy subscale (6.37; 3.15) | *r* = .21 |
|  |  |  |  |  | Sibling Relationship Questionnaire SRQ, prosocial behavior subscale (8.38; 2.76) | *r* = .20 |
|  |  |  |  |  | Sibling Relationship Questionnaire SRQ, companionship subscale (9.96; 3.06) | *r* = .23 |
|  |  |  |  |  | Sibling Relationship Questionnaire SRQ, similarity subscale (7.60; 2.87) | *r* = .15 |
|  |  |  |  |  | Sibling Relationship Questionnaire SRQ, admiration of sibling subscale (9.77; 3.11) | *r* = .07 |
|  |  |  |  |  | Sibling Relationship Questionnaire SRQ, admiration by sibling subscale (9.58; 3.08) | *r* = .08 |
|  |  |  |  |  | Sibling Relationship Questionnaire SRQ, affection subscale (11.20; 3.09) | *r* = .19 |
|  |  |  |  | Dyadic Adjustment Scale DAS, mother-rated (115.53; 14.48) | Sibling Relationship Questionnaire SRQ, intimacy subscale (6.37; 3.15) | *r* = .08 |
|  |  |  |  |  | Sibling Relationship Questionnaire SRQ, prosocial behavior subscale (8.38; 2.76) | *r* = .09 |
|  |  |  |  |  | Sibling Relationship Questionnaire SRQ, companionship subscale (9.96; 3.06) | *r* = .24 |
|  |  |  |  |  | Sibling Relationship Questionnaire SRQ, similarity subscale (7.60; 2.87) | *r* = -.03 |
|  |  |  |  |  | Sibling Relationship Questionnaire SRQ, admiration of sibling subscale (9.77; 3.11) | *r* = .00 |
|  |  |  |  |  | Sibling Relationship Questionnaire SRQ, admiration by sibling subscale (9.58; 3.08) | *r* = .10 |
|  |  |  |  |  | Sibling Relationship Questionnaire SRQ, affection subscale (11.20; 3.09) | *r* = .19 |
| Ingoldsby et al. | 2001 | USA | *N* = 117 boys | Conflict Tactics Scale CTS, verbal aggression and physical aggression subscales at Time 1 (24.58; 21.47) | Sibling Conflict Coding System, destructiveness standardized score at Time 2 (-.006; .94) | *r* = .07 |
| Iturralde et al. | 2013 | USA | *N* = 52 sibling pairs | Children’s Perception of Interparental Conflict Questionnaire CPIC, conflict properties subscale (33.12; 8.36) | Observer rating of positivity (1.97; .45) | *r* = .18^c^ |
|  |  |  |  |  | Observer rating of negativity (0.70; .55) | *r* = -.01 |

**Table S2 continued**

| **Authors** | **Publication year** | **Country of study** | **Sample^a^** | **Interparental relationship measure  (M; SD)^b^** | **Sibling relationship measure  (M; SD)^b^** | **Interparental-sibling association** |
| --- | --- | --- | --- | --- | --- | --- |
| Lauretti | 2001 | USA | *N* = 40 families | Aggregated observer ratings of intimacy of communication (3.19; .56), warmth between partners (3.14; .75), overt conflict (2.87; .90), problem-solving (3.14; .60), power (3.53; .79), individuation/autonomy (3.35; .59) and self-report measures of mothers’ (6.04; 2.28) and fathers’ (6.24; 1.97) satisfaction with discussions | Observer rating of positive bids (1.73; 1.45) | *r* = -.18 |
|  |  |  |  |  | Observer rating of negative bids (0.43; .93) | *r* = .08^c^ |
|  |  |  |  |  | Observer ratings of positive affect (1.95; 3.52) and prosocial behavior (2.12, 1.20) | *r* = .21 |
|  |  |  |  |  | Observer ratings of negative affect (1.60, 1.35), overt conflict (1.35; 1.00), behavioral competition (1.65; 1.05) and dominance (1.78; 1.39) | *r* = -.40^c^ |
| Lindsey et al. | 2006 | USA | *N* = 113 nondivorced families | O'Leary-Porter-Scale OPS (28.66; 6.78) | Conflict Tactics Scale CTS, sibling conflict resolution computed as reasoning subscale minus verbal aggression subscale (1.67; 1.22) | *r* = .19^c^ |
|  |  |  | *N* = 60 divorced families | O'Leary-Porter-Scale OPS (31.53; 4.99) | Conflict Tactics Scale CTS, sibling conflict resolution computed as reasoning subscale minus verbal aggression subscale (1.67; 1.13) | *r* = -.00^c,d^ |
| Liu | 2006 | USA | *N* = 64 families | Domestic Conflict Inventory DCI  (0.53; 1.17), mother to father | Sibling Relationship Questionnaire Child/Parent SRQ-C/P (3.49; .60) | *r* = -.01 |
|  |  |  |  | Domestic Conflict Inventory DCI  (0.67; 2.16), father to mother | Sibling Relationship Questionnaire Child/Parent SRQ-C/P (3.49; .60) | *r* = -.14 |
|  |  |  |  | Children’s Perception of Interparental Conflict Scale CPIC, conflict resolution subscale (9.25; 3.13) | Sibling Relationship Questionnaire Child/Parent SRQ-C/P (3.49; .60) | *r* = -.11^c^ |
| MacKinnon | 1989 | USA | *N* = 48 sibling pairs from divorced families | Family History Inventory FHI | Family History Inventory FHI | *r* = .33 |
|  |  |  |  |  | Observer rating of positive interactions | *r* = .35 |
|  |  |  |  |  | Observer rating of negative interactions | *r* = .42^c^ |
|  |  |  | *N* = 48 sibling pairs from married families |  | Family History Inventory FHI | *r* = .51 |
|  |  |  |  |  | Observer rating of positive interactions | *r* = .36 |
|  |  |  |  |  | Observer rating of negative interactions | *r* = .39^c^ |

**Table S2 continued**

| **Authors** | **Publication year** | **Country of study** | **Sample^a^** | **Interparental relationship measure  (M; SD)^b^** | **Sibling relationship measure  (M; SD)^b^** | **Interparental-sibling association** |
| --- | --- | --- | --- | --- | --- | --- |
| Masarik & Rogers | 2019 | USA | *N* = 351 adolescents | Observer rating of romantic relationship hostility (4.01; 1.02) | Unpublished measure of perceived warmth | *r* = .27^c^ |
|  |  |  | *N* = 106 girls with a sister | Observer rating of romantic relationship hostility |  | *r* = .30^c^ |
|  |  |  | *N* = 77 boys with a brother |  |  | *r* = .11^c^ |
|  |  |  | *N* = 86 girls with a brother |  |  | *r* = .32^c^ |
|  |  |  | *N* = 82 boys with a sister |  |  | *r* = .45^c^ |
| McGuire et al. | 1996 | USA | *n* = 19 hostile (high hostility, low positivity) and *n* = 15 affect-intense (high hostility, high positivity) siblings | Relationships Questionnaire, love subscale (hostile = 71.5); (affect-intense = 75.8) | Sibling Relationship Inventory SRI, positivity subscale (hostile = 2.21; .53); (affect-intense = 3.35; .46) | *F*(1,32) = 7.54 |
|  |  |  |  | Aspects of Marital Life Scale, satisfaction subscale (hostile = 69.3); (affect-intense = 77.3) |  | *F*(1,32) = 6.49 |
| McLean | 2006 | USA | *N* = 73 adolescents | Children’s Perception of Interparental Conflict Scale CPIC (16; 9.70) | Sibling Relationship Questionnaire SRQ-R (3.3; .70) | *r* = -.14^c^ |
|  |  |  |  |  | Sibling Relationship Questionnaire SRQ-R (2.9; .87) | *r* = .17 |
| Miller et al. | 2012 | USA | *N* = 150 children | Conflict Tactics Scale CTS, mild & severe physical assault subscales (15.67; 45.46) | Sibling Social Behavior Scale SSBS  (1.82; .60) | *r* = .19 |
| Nagel | 1996 | USA | *N* = 61 families | Unpublished measure of marital harmony  (4.11; .69) | Unpublished measure of sibling harmony (5.36; 2.07) | *r* = .20 |
|  |  |  |  |  | Unpublished measure of sibling conflict (4.83; 1.88) | *r* = .07^c^ |
|  |  |  |  |  | Observer rating of negative comments (2.47; 4.20) | *r* = .42^c^ |
|  |  |  |  |  | Observer rating of positive engagement 2.50 (0.57) | *r* = -.17 |

**Table S2 continued**

| **Authors** | **Publication year** | **Country of study** | **Sample^a^** | **Interparental relationship measure  (M; SD)^b^** | **Sibling relationship measure  (M; SD)^b^** | **Interparental-sibling association** |
| --- | --- | --- | --- | --- | --- | --- |
|  |  |  |  | Unpublished measure of marital conflict  (2.05; .80) | Unpublished measure of sibling harmony (5.36; 2.07) | *r* = -.03^c^ |
|  |  |  |  |  | Unpublished measure of sibling conflict (4.83; 1.88) | *r* = -.06 |
|  |  |  |  |  | Observer rating of negative comments (2.47; 4.20) | *r* = .41 |
|  |  |  |  |  | Observer rating of positive engagement 2.50 (0.57) | *r* = .06^c^ |
|  |  |  |  | Observer rating of negative comments  (1.45; 2.56) | Unpublished measure of sibling harmony (5.36; 2.07) | *r* = -.09^c^ |
|  |  |  |  |  | Unpublished measure of sibling conflict (4.83; 1.88) | *r* = -.16 |
|  |  |  |  |  | Observer rating of negative comments (2.47; 4.20) | *r* = .00 |
|  |  |  |  |  | Observer rating of positive engagement 2.50 (0.57) | *r* = .23^c^ |
|  |  |  |  | Observer rating of marital harmony  (3.67; .92) | Unpublished measure of sibling harmony (5.36; 2.07) | *r* = -.18 |
|  |  |  |  |  | Unpublished measure of sibling conflict (4.83; 1.88) | *r* = -.30^c^ |
|  |  |  |  |  | Observer rating of negative comments (2.47; 4.20) | *r* = .10^c^ |
|  |  |  |  |  | Observer rating of positive engagement 2.50 (0.57) | *r* = .14 |
|  |  |  |  | Children’s Perceptions of Interparental Conflict Scale CPIC (1.71; .57) | Unpublished measure of sibling harmony (5.36; 2.07) | *r* = .09^c^ |
|  |  |  |  |  | Unpublished measure of sibling conflict (4.83; 1.88) | *r* = .31 |
|  |  |  |  |  | Observer rating of negative comments (2.47; 4.20) | *r* = .16 |
|  |  |  |  |  | Observer rating of positive engagement 2.50 (0.57) | *r* = .09^c^ |

**Table S2 continued**

| **Authors** | **Publication year** | **Country of study** | **Sample^a^** | **Interparental relationship measure  (M; SD)^b^** | **Sibling relationship measure  (M; SD)^b^** | **Interparental-sibling association** |
| --- | --- | --- | --- | --- | --- | --- |
| Odudu | 2018 | USA | *N* = 101 families | Network of Relationships Inventory NRI, positivity subscale (3.52; .86) | Observer rating of constructive conflict  (3.25; .58) | *r* = .00 |
|  |  |  |  |  | Observer Rating of destructive conflict  (1.11; .28) | *r* = .07^c^ |
|  |  |  |  | Network of Relationships Inventory NRI, negativity subscale (1.98; .66) | Observer Rating of constructive conflict (3.25; .58) | *r* = -.10^c^ |
|  |  |  |  |  | Observer Rating of destructive conflict  (1.11; .28) | *r* = .21 |
|  |  |  |  | Network of Relationships Inventory NRI, positivity subscale (3.52; .86) | Network of Relationships Inventory NRI, positivity subscale at time 1 (3.30; .77) | *r* = .18 |
|  |  |  |  |  | Network of Relationships Inventory NRI, positivity subscale at time 2 (3.07; .87) | *r* = -.10 |
|  |  |  |  |  | Network of Relationships Inventory NRI, negativity subscale at time 1 (2.85; .94) | *r* = .04^c^ |
|  |  |  |  |  | Network of Relationships Inventory NRI, negativity subscale at time 2 (2.86; 1.04) | *r* = .22^c^ |
|  |  |  |  | Network of Relationships Inventory NRI, negativity subscale (1.98; .66) | Network of Relationships Inventory NRI, positivity subscale at time 1 (3.30; .77) | *r* = .04^c^ |
|  |  |  |  |  | Network of Relationships Inventory NRI, positivity subscale at time 2 (3.07; .87) | *r* = -.06^c^ |
|  |  |  |  |  | Network of Relationships Inventory NRI, negativity subscale at time 1 (2.85; .94) | *r* = .12 |
|  |  |  |  |  | Network of Relationships Inventory NRI, negativity subscale at time 2 (2.86; 1.04) | *r* = .04 |
| Piotrowski et al. | 2017 | Canada | *N* = 47 sibling dyads and their mothers | Conflict Tactics Scale CTS | Child Behavior Checklist, Externalizing Disorder Scale CBCL | *r* = .53 |
|  |  |  |  | Violence Exposure Scale for Children |  | *r* = .62 |

**Table S2 continued**

| **Authors** | **Publication year** | **Country of Study** | | **Sample^a^** | | **Interparental relationship measure  (M; SD)^b^** | | **Sibling relationship measure  (M; SD)^b^** | | **Interparental-sibling association** |
| --- | --- | --- | --- | --- | --- | --- | --- | --- | --- | --- |
| Query | 2000 | USA | | *N* = 69 families | | Short Marital Adjustment Test SMAT, mother-rated (119.09; 24.85) | | Sibling Relationship Questionnaire SRQ, warmth subscale (3.52; .60) | | *r* = .35 |
|  |  |  | |  | |  | | Sibling Relationship Questionnaire SRQ, conflict subscale (2.82; .71) | | *r* = .15^c^ |
|  |  |  | |  | |  | | Sibling Relationship Questionnaire SRQ, aggression subscale (102.03; 89.09) | | *r* = .23^c^ |
|  |  |  | | *N* = 66 families | | Short Marital Adjustment Test SMAT, father-rated (111.82; 25.94) | | Sibling Relationship Questionnaire SRQ, warmth subscale (3.41; .58) | | *r* = .08 |
|  |  |  | |  | |  | | Sibling Relationship Questionnaire SRQ, conflict subscale (2.84; .61) | | *r* = -.23^c^ |
|  |  |  | |  | |  | | Sibling Relationship Questionnaire SRQ, aggression subscale (104.68; 96.07) | | *r* = .05^c^ |
| Reese-Weber | 2000 | USA | | *N* = 89 adolescents | | Conflict-Resolution Behavior Questionnaire, attack subscale (21.9; 12.0) | | Conflict-Resolution Behavior Questionnaire, attack subscale (33.7; 11.0) | | *r* = .28 |
|  |  |  | |  | | Conflict-Resolution Behavior Questionnaire, compromise subscale (25.3; 6.6) | | Conflict-Resolution Behavior Questionnaire, compromise subscale (19.4; 7.6) | | *r* = .34 |
| Rinaldi & Howe | 2003 | Canada | | *N* = 60 families | | Conflict Tactics Scale CTS | | Sibling Relationship Questionnaire, conflict subscale (3.23; .75) | | *r* = .26 |
|  |  |  | |  | |  | |  | | *r* = .25 |
|  |  |  | |  | |  | |  | | *r* = .20 |
|  |  |  | |  | |  | |  | | *r* = .11 |
| Ruff | 2012 | USA | *N* = 244 families | | Relationship Assessment Battery RELATE at time 2, mother- (19.46; 3.98) & father-rated (18.97; 4.10) | | Sibling Relationship Inventory SRI, affection subscale at time 2 (18.26; 5.20) | | *r* = .06^c^ | |
|  |  |  |  | |  |  | SRI, hostility subscale at time 2 (13.65; 3.63) | | *r* = .16 | |
|  |  |  |  | |  | | SRI, affection subscale at time 3 (17.91; 4.92) | | *r* = .04^c^ | |
|  |  |  |  | |  | | SRI, hostility subscale at time 3 (13.64; 3.80) | | *r* = .09 | |
|  |  |  |  | |  | | SRI, affection subscale at time 4 (17.68; 5.08) | | *r* = .10^c^ | |
|  |  |  |  | |  | | SRI, hostility subscale at time 4 (12.91; 3.54) | | *r* = .16 | |

**Table S2 continued**

| **Authors** | **Publication year** | **Country of Study** | **Sample^a^** | **Interparental relationship measure  (M; SD)^b^** | **Sibling relationship measure  (M; SD)^b^** | **Interparental-sibling association** |
| --- | --- | --- | --- | --- | --- | --- |
| Ruff et al. | 2018 | USA | *N* = 400 families | Relationship Assessment Battery RELATE at time 2, marital conflict subscale, mother-rated (2.48; .51) | Sibling Relationship Inventory SRI, affection subscale at time 2 (3.03; .89) | *r* = .06^c^ |
|  |  |  |  |  | SRI, affection subscale at time 3 (3.04; .87) | *r* = -.01^c^ |
|  |  |  |  |  | SRI, affection subscale at time 4 (2.96; .86) | *r* = .06^c^ |
|  |  |  |  |  | SRI, hostility subscale at time 2 (2.75; .79) | *r* = .19 |
|  |  |  |  |  | SRI, hostility subscale at time 3 (2.74; .81) | *r* = .09 |
|  |  |  |  |  | SRI, hostility subscale at time 4 (2.57; .76) | *r* = .16 |
|  |  |  |  | Relationship Assessment Battery RELATE at time 2, marital conflict subscale, father-rated (2.40; .51) | SRI, affection subscale at time 2 (3.03; .89) | *r* = .07^c^ |
|  |  |  |  |  | SRI, affection subscale at time 3 (3.04; .87) | *r* = -.01^c^ |
|  |  |  |  |  | SRI, affection subscale at time 4 (2.96; .86) | *r* = .08^c^ |
|  |  |  |  |  | SRI, hostility subscale at time 2 (2.75; .79) | *r* = .22 |
|  |  |  |  |  | SRI, hostility subscale at time 3 (2.74; .81) | *r* = .11 |
|  |  |  |  |  | SRI, hostility subscale at time 4 (2.57; .76) | *r* = .14 |
| Scrimgeour | 2015 | USA | *N* = 70 families | Love and Respect Scale (4.19; .57) | Sibling Inventory of Behavior, positive involvement subscale, older sibling  (3.78; .58) | *r* = .21 |
|  |  |  |  |  | Observer rating of prosocial behavior, helping, older sibling (2.70; 3.50) | *r* = .07 |
|  |  |  |  |  | Observer rating of prosocial behavior, sharing, older sibling (2.24; 2.87) | *r* = .07 |
|  |  |  |  |  | Sibling Inventory of Behavior, positive involvement subscale, younger sibling  (3.91; .44) | *r* = .31 |
|  |  |  |  |  | Observer rating of prosocial behavior, helping, younger sibling (0.34; .78) | *r* = .16 |
|  |  |  |  |  | Observer rating of prosocial behavior, sharing, younger sibling (1.98; 2.49) | *r* = .10 |

**Table S2 continued**

| **Authors** | **Publication year** | **Country of Study** | **Sample^a^** | **Interparental relationship measure  (M; SD)^b^** | **Sibling relationship measure  (M; SD)^b^** | **Interparental-sibling association** |
| --- | --- | --- | --- | --- | --- | --- |
| Senguttuvan | 2014 | USA | *N* = 690 families | Unpublished measure of marital happiness (8.41; 1.66) | Unpublished measure of sibling intimacy (3.87; 1.07) | *r* = .06 |
|  |  |  |  | Unpublished measure of marital conflict  (2.18; .72) | Unpublished measure of sibling conflict (3.12; 1.12) | *r* = .00^c^ |
|  |  |  |  | Unpublished measure of marital happiness (8.41; 1.66) | Unpublished measure of sibling intimacy (3.87; 1.07) | *r* = -.02^c^ |
|  |  |  |  | Unpublished measure of marital conflict  (2.18; .72) | Unpublished measure of sibling conflict (3.12; 1.12) | *r* = -.01 |
| Sigda | 1999 | USA | *N* = 50 children and their families | CPIC, intensity & frequency subscales  (1.95; .51) | Sibling Relationship Questionnaire SRQ, warmth subscale (2.79; .69) | *r* = .10^c^ |
|  |  |  |  |  | Sibling Relationship Questionnaire SRQ, negativity subscale (2.72; .61) | *r* = .08 |
| Soliday | 1996 | USA | *N* = 25 families expecting a second child | Dyadic Adjustment Scale Short Form DAS-SF, mother-rated (22.25; 3.47) | Observer rating of positive engagement | *r* = .31 |
|  |  |  |  |  | Observer rating of negative engagement | *r* = .36^c^ |
|  |  |  |  | Dyadic Adjustment Scale Short Form DAS-SF, mother-rated (22.12; 3.19) | Observer rating of positive engagement | *r* = .43 |
|  |  |  |  |  | Observer rating of negative engagement | *r* = .46^c^ |
| Stocker et al. | 1997 | USA | *N* = 64 children and their mothers | Dyadic Adjustment Scale DAS, marital satisfaction | Sibling Relationship Questionnaire SRQ, warmth subscale | *r* = .11 |
|  |  |  |  |  | Sibling Relationship Questionnaire SRQ, hostility subscale | *r* = .33^c^ |
|  |  |  |  |  | Sibling Relationship Questionnaire SRQ, rivalry subscale | *r* = .18^c^ |
|  |  |  |  | Dyadic Adjustment Scale DAS, marital affectional expression | Sibling Relationship Questionnaire SRQ, warmth subscale | *r* = .16 |
|  |  |  |  |  | Sibling Relationship Questionnaire SRQ, hostility subscale | *r* = .31^c^ |
|  |  |  |  |  | Sibling Relationship Questionnaire SRQ, rivalry subscale | *r* = .28^c^ |

**Table S2 continued**

| **Authors** | **Publication year** | **Country of Study** | **Sample^a^** | **Interparental relationship measure  (M; SD)^b^** | **Sibling relationship measure  (M; SD)^b^** | **Interparental-sibling association** |
| --- | --- | --- | --- | --- | --- | --- |
| Stocker & Youngblade | 1999 | USA | *N* = 136 families | O'Leary-Porter Scale of Marital Conflict OPS, mother-rated (1.07; .51), father-rated (1.06; .49) & Marital Interaction Coding System, mothers (1.50; .84), fathers (1.39; .69) | Sibling Relationship Questionnaire SRQ, warmth subscale (2.18; .43) | *r* = .17^c^ |
|  |  |  |  |  | Sibling Relationship Questionnaire SRQ, conflict subscale (2.16; .50) | *r* = .25 |
|  |  |  |  |  | Sibling Relationship Questionnaire SRQ, rivalry subscale (1.39; .51) | *r* = .26 |
| Tucker et al. | 2014 | USA | *N* = 866 children with no victimization and  *N* = 802 children with  common victimization | Children’s’ Perception of Interparental Conflict CPIC, conflict properties subscale (2.62; .96) | Juvenile Victimization Questionnaire JVQ | OR = .99 |
|  |  |  | *N* = 866 children with no victimization and  *N* = 57 children with severe victimization |  | Juvenile Victimization Questionnaire, severe physical assault | OR = 1.43 |
| Tucker et al. | 2020 | USA | *N* = 2659 children and adolescents | Children’s’ Perception of Interparental Conflict CPIC, conflict properties subscale | Juvenile Victimization Questionnaire JVQ, no victimization vs. sibling victimization | OR = .93 |
| Volling et al. | 2002 | USA | *N* = 60 families | Intimate Relations Scale, marital love & maintenance subscales (103.35; 12.27) | Sibling inventory of Behavior SIB, older sibling positive involvement, mother-rated (50.65; 8.30) | *r* = .08 |
|  |  |  |  |  | SIB, older sibling rivalry, mother-rated (31.59; 6.29) | *r* = -.15^c^ |
|  |  |  |  |  | SIB, younger sibling positive involvement, mother-rated (28.70; 4.70) | *r* = .23 |
|  |  |  |  |  | SIB, younger sibling rivalry, mother-rated (15.51; 3.51) | *r* = -.06^c^ |
|  |  |  |  |  | SIB, older sibling positive involvement, father-rated (48.93; 8.49) | *r* = .25 |
|  |  |  |  |  | SIB, older sibling rivalry, father-rated  (30.87; 6.37) | *r* = -.01^c^ |
|  |  |  |  |  | SIB, younger sibling positive involvement, father-rated (27.68; 3.89) | *r* = .13 |
|  |  |  |  |  | SIB, younger sibling rivalry, father-rated (14.12; 3.16) | *r* = .06^c^ |

**Table S2 continued**

| **Authors** | **Publication year** | **Country of study** | **Sample^a^** | **Interparental relationship measure  (M; SD)^b^** | **Sibling relationship measure  (M; SD)^b^** | **Interparental-sibling association** |
| --- | --- | --- | --- | --- | --- | --- |
|  |  |  |  | Intimate Relations Scale, marital conflict & feelings of ambivalence subscales  (35.00; 11.85) | SIB, older sibling positive involvement, mother-rated (50.65; 8.30) | *r* = .19^c^ |
|  |  |  |  |  | SIB, older sibling rivalry, mother-rated (31.59; 6.29) | *r* = .04 |
|  |  |  |  |  | SIB, younger sibling positive involvement, mother-rated (28.70; 4.70) | *r* = .20^c^ |
|  |  |  |  |  | SIB, younger sibling rivalry, mother-rated (15.51; 3.51) | *r* = .05 |
|  |  |  |  |  | SIB, older sibling positive involvement, father-rated (48.93; 8.49) | *r* = .32^c^ |
|  |  |  |  |  | SIB, older sibling rivalry, father-rated  (30.87; 6.37) | *r* = .21 |
|  |  |  |  |  | SIB, younger sibling positive involvement, father-rated (27.68; 3.89) | *r* = .20^c^ |
|  |  |  |  |  | SIB, younger sibling rivalry, father-rated (14.12; 3.16) | *r* = .19 |
| Weaver-Graham | 1998 | USA | *N* = 28 depressed children with a depressed parent | Psychosocial Schedule for School Age Children PSS, warmth subscale (1.72; 1.03) | Psychosocial Schedule for School Age Children PSS, warmth subscale (1.97; 1.19) | *r* = .30 |
|  |  |  | *N* = 34 children with a depressed parent | Psychosocial Schedule for School Age Children PSS, warmth subscale (1.71; .86) | Psychosocial Schedule for School Age Children PSS, warmth subscale (1.63; .79) | *r* = .13 |
|  |  |  | *N* = 39 nondepressed children | Psychosocial Schedule for School Age Children PSS, warmth subscale (1.26; .68) | Psychosocial Schedule for School Age Children PSS, warmth subscale (1.25; .54) | *r* = .47 |

**Table S2 continued**

| **Authors** | **Publication year** | **Country of study** | **Sample^a^** | **Interparental relationship measure  (M; SD)^b^** | **Sibling relationship measure  (M; SD)^b^** | **Interparental-sibling association** |
| --- | --- | --- | --- | --- | --- | --- |
| Yu & Gamble | 2008 | USA, Taiwan | *N* = 130 mothers and their preschool-aged child | Short Marital Adjustment Test SMAT (35.40; 23.01) | Parental Expectations and Perceptions of Children's Sibling Relationships Questionnaire PEPC-SRQ, warmth subscale (3.98; .589) | *r* = .15 |
|  |  |  |  |  | Parental Expectations and Perceptions of Children's Sibling Relationships Questionnaire PEPC-SRQ, agonism subscale (3.00; .77) | *r* = -.13^c^ |
|  |  |  |  |  | Parental Expectations and Perceptions of Children's Sibling Relationships Questionnaire PEPC-SRQ, competition subscale (3.98; .87) | *r* = -.15^c^ |

*Note.* Blank table fields represent repetitions of the row above. ^a^For longitudinal studies, sample characteristics of baseline assessments (first time points) are reported. ^b^Means and standard deviations for relationship measures are only reported for primary studies that originally reported them. ^c^Signs were switched for effects, for which the dimensions (positive/negative) of interparental and sibling relationship qualities differed in direction. ^d^Values of .00 were imputed because authors reported that no significant association was found.
