# Supplementary material for: A systematic review and meta-analysis of the associations between interparental and sibling relationships: Positive or negative?
Source: PLoS One. 2021 Sep 28;16(9):e0257874. doi: 10.1371/journal.pone.0257874 (PMC8478168; doi:10.1371/journal.pone.0257874)
Supplement: S3 Table — (DOCX) [file pone.0257874.s004.docx]

**S3 Table**

*Coded moderators of included studies (k = 47)*

| **Authors** | **[A]** | **[B]** | **[C]** | **[D]** | **[E]** | **[F]** | **[G]** | **[H]** | **[I]** | **[J]** | **[K]** | **[L]** | **[M]** | **[N]** |
| --- | --- | --- | --- | --- | --- | --- | --- | --- | --- | --- | --- | --- | --- | --- |
| Beyers Carlson (2018) | 1 | 1 | 1 | 3 | 1 | 2.58 | 54.8 | 2 | 1 | 1 | 1 | 2 | 2 | 4 |
|  | 2 | 1 | 1 | 3 | 1 | 2.58 | 54.8 | 2 | 1 | 1 | 1 | 2 | 2 | 4 |
| Brockman (1994) | 1 | 1 | 1 | 2 | 3 | -99 | 70 | 2 | 1 | 1 | 1 | 1 | 2 | 4 |
| Brody et al. (1987) | 1 | 1 | 1 | 3 | 2 | -99 | 48 | 1 | 1 | 1 | 1 | 1 | 2 | 1 |
|  | 1 | 1 | 2 | 3 | 2 | -99 | 48 | 1 | 1 | 1 | 1 | 1 | 2 | 1 |
|  | 1 | 1 | 1 | 3 | 2 | -99 | 48 | 1 | 1 | 1 | 1 | 1 | 2 | 1 |
|  | 1 | 1 | 2 | 3 | 2 | -99 | 48 | 1 | 1 | 1 | 1 | 1 | 2 | 1 |
| Brody et al. (1994) | 1 | 1 | 1 | 2 | 3 | -99 | 55 | 1 | 2 | 1 | 1 | 2 | 2 | 1 |
|  | 1 | 1 | 2 | 2 | 3 | -99 | 55 | 1 | 2 | 1 | 1 | 2 | 2 | 1 |
|  | 1 | 1 | 1 | 2 | 3 | -99 | 55 | 1 | 2 | 1 | 1 | 2 | 2 | 1 |
|  | 1 | 1 | 2 | 2 | 3 | -99 | 55 | 1 | 2 | 1 | 1 | 2 | 2 | 1 |
|  | 1 | 1 | 1 | 2 | 3 | -99 | 55 | 1 | 2 | 1 | 1 | 2 | 2 | 1 |
|  | 1 | 1 | 2 | 2 | 3 | -99 | 55 | 1 | 2 | 1 | 1 | 2 | 2 | 1 |
|  | 1 | 1 | 1 | 2 | 3 | -99 | 55 | 1 | 2 | 1 | 1 | 2 | 2 | 1 |
|  | 1 | 1 | 2 | 2 | 3 | -99 | 55 | 1 | 2 | 1 | 1 | 2 | 2 | 1 |
| Button & Gealt (2010) | 2 | 2 | 2 | 2 | 4 | -99 | 46 | 3 | 3 | 3 | 1 | 1 | 2 | 1 |
| Caya (2001) | 2 | 1 | 1 | 2 | 3 | 4.64 | 46.1 | 2 | 1 | 3 | 1 | 1 | 2 | 4 |
| Conners (1999) | 1 | 1 | 1 | 1 | 3 | 2.33 | 0 | 2 | 3 | 1 | 1 | 1 | 2 | 4 |
|  | 1 | 1 | 2 | 1 | 3 | 2.33 | 0 | 2 | 3 | 1 | 1 | 1 | 2 | 4 |
|  | 1 | 1 | 1 | 2 | 3 | 2.33 | 0 | 2 | 3 | 1 | 1 | 1 | 2 | 4 |
|  | 1 | 1 | 2 | 2 | 3 | 2.33 | 0 | 2 | 3 | 1 | 1 | 1 | 2 | 4 |
|  | 1 | 1 | 1 | 1 | 3 | 2.33 | 0 | 2 | 3 | 1 | 1 | 1 | 2 | 4 |
|  | 1 | 1 | 2 | 1 | 3 | 2.33 | 0 | 2 | 3 | 1 | 1 | 1 | 2 | 4 |
|  | 1 | 1 | 1 | 2 | 3 | 2.33 | 0 | 2 | 3 | 1 | 1 | 1 | 2 | 4 |
|  | 1 | 1 | 2 | 2 | 3 | 2.33 | 0 | 2 | 3 | 1 | 1 | 1 | 2 | 4 |
| Dantchev & Wolke (2019) | 2 | 1 | 2 | 2 | 3 | -99 | 46.6 | 2 | 3 | 3 | 1 | 2 | 2 | 1 |
|  | 2 | 1 | 2 | 2 | 3 | -99 | 46.6 | 2 | 3 | 3 | 1 | 2 | 2 | 1 |
| Davies et al. (2018) | 2 | 3 | 1 | 1 | 3 | 3.32 | 51 | 2 | 3 | 3 | 1 | 1 | 2 | 1 |
| Dawson et al. (2014) | 2 | 1 | 1 | 2 | 2 | 2.17 | -99 | 2 | 1 | 1 | 1 | 1 | 2 | 1 |
|  | 2 | 1 | 1 | 2 | 2 | 2.17 | -99 | 2 | 1 | 1 | 1 | 1 | 2 | 1 |

**Table S3 continued**

| **Authors** | **[A]** | **[B]** | **[C]** | **[D]** | **[E]** | **[F]** | **[G]** | **[H]** | **[I]** | **[J]** | **[K]** | **[L]** | **[M]** | **[N]** |
| --- | --- | --- | --- | --- | --- | --- | --- | --- | --- | --- | --- | --- | --- | --- |
| Dekovic & Buist (2005) | 1 | 1 | 1 | 2 | 3 | -99 | 50 | 2 | 3 | 1 | 1 | 1 | 2 | 1 |
| Dunn et al. (1999) | 1 | 1 | 1 | 1 | 2 | -99 | 51.6 | 2 | 1 | 1 | 1 | 2 | 2 | 1 |
|  | 1 | 1 | 2 | 1 | 2 | -99 | 51.6 | 2 | 1 | 1 | 1 | 2 | 2 | 1 |
|  | 1 | 1 | 1 | 1 | 2 | -99 | 51.6 | 2 | 1 | 1 | 1 | 2 | 2 | 1 |
|  | 1 | 1 | 2 | 1 | 2 | -99 | 51.6 | 2 | 1 | 1 | 1 | 2 | 2 | 1 |
|  | 2 | 1 | 1 | 1 | 2 | -99 | 51.6 | 2 | 1 | 1 | 1 | 2 | 2 | 1 |
|  | 2 | 1 | 2 | 1 | 2 | -99 | 51.6 | 2 | 1 | 1 | 1 | 2 | 2 | 1 |
|  | 2 | 1 | 1 | 1 | 2 | -99 | 51.6 | 2 | 1 | 1 | 1 | 2 | 2 | 1 |
|  | 2 | 1 | 2 | 1 | 2 | -99 | 51.6 | 2 | 1 | 1 | 1 | 2 | 2 | 1 |
| Erel et al. (1998) | 2 | 1 | 2 | 3 | 2 | 2.42 | -99 | 1 | 1 | 1 | 1 | 1 | 2 | 1 |
|  | 2 | 1 | 1 | 3 | 2 | 2.42 | -99 | 1 | 1 | 1 | 1 | 1 | 2 | 1 |
|  | 1 | 1 | 2 | 3 | 2 | 2.42 | -99 | 1 | 1 | 1 | 1 | 1 | 2 | 1 |
|  | 1 | 1 | 1 | 3 | 2 | 2.42 | -99 | 1 | 1 | 1 | 1 | 1 | 2 | 1 |
| Grych et al. (2004) | 2 | 2 | 1 | 2 | 4 | -99 | 39.9 | 3 | 3 | 3 | 1 | 1 | 2 | 1 |
| Guinn et al. (2012) | 1 | 1 | 1 | 2 | 3 | -99 | 100 | 3 | 3 | 1 | 1 | 1 | 2 | 4 |
|  | 1 | 1 | 1 | 2 | 3 | -99 | 0 | 3 | 3 | 1 | 1 | 1 | 2 | 4 |
|  | 1 | 1 | 2 | 2 | 3 | -99 | 100 | 3 | 3 | 1 | 1 | 1 | 2 | 4 |
|  | 1 | 1 | 2 | 2 | 3 | -99 | 0 | 3 | 3 | 1 | 1 | 1 | 2 | 4 |
|  | 1 | 1 | 1 | 2 | 3 | -99 | 100 | 3 | 3 | 1 | 1 | 1 | 2 | 4 |
|  | 1 | 1 | 1 | 2 | 3 | -99 | 0 | 3 | 3 | 1 | 1 | 1 | 2 | 4 |
|  | 1 | 1 | 2 | 2 | 3 | -99 | 100 | 3 | 3 | 1 | 1 | 1 | 2 | 4 |
|  | 1 | 1 | 2 | 2 | 3 | -99 | 0 | 3 | 3 | 1 | 1 | 1 | 2 | 4 |

**Table S3 continued**

| **Authors** | **[A]** | **[B]** | **[C]** | **[D]** | **[E]** | **[F]** | **[G]** | **[H]** | **[I]** | **[J]** | **[K]** | **[L]** | **[M]** | **[N]** |
| --- | --- | --- | --- | --- | --- | --- | --- | --- | --- | --- | --- | --- | --- | --- |
| Haj-Yahia & Abdo-Kaloti (2003) | 2 | 2 | 2 | 2 | 4 | -99 | 49.8 | 3 | 3 | 3 | 1 | 1 | 2 | 1 |
|  | 2 | 2 | 2 | 2 | 4 | -99 | 49.8 | 3 | 3 | 3 | 1 | 1 | 2 | 1 |
|  | 2 | 2 | 2 | 2 | 4 | -99 | 49.8 | 3 | 3 | 3 | 1 | 2 | 2 | 1 |
|  | 2 | 2 | 2 | 2 | 4 | -99 | 49.8 | 3 | 3 | 3 | 1 | 2 | 2 | 1 |
|  | 2 | 2 | 2 | 2 | 4 | -99 | 49.8 | 3 | 3 | 3 | 1 | 1 | 2 | 1 |
|  | 2 | 2 | 2 | 2 | 4 | -99 | 49.8 | 3 | 3 | 3 | 1 | 1 | 2 | 1 |
|  | 2 | 2 | 2 | 2 | 4 | -99 | 49.8 | 3 | 3 | 3 | 1 | 2 | 2 | 1 |
|  | 2 | 2 | 2 | 2 | 4 | -99 | 49.8 | 3 | 3 | 3 | 1 | 2 | 2 | 1 |
|  | 2 | 2 | 2 | 2 | 4 | -99 | 49.8 | 3 | 3 | 3 | 1 | 1 | 2 | 1 |
|  | 2 | 2 | 2 | 2 | 4 | -99 | 49.8 | 3 | 3 | 3 | 1 | 1 | 2 | 1 |
|  | 2 | 2 | 2 | 2 | 4 | -99 | 49.8 | 3 | 3 | 3 | 1 | 1 | 2 | 1 |
|  | 2 | 2 | 2 | 2 | 4 | -99 | 49.8 | 3 | 3 | 3 | 1 | 1 | 2 | 1 |
|  | 2 | 2 | 2 | 2 | 4 | -99 | 49.8 | 3 | 3 | 3 | 1 | 1 | 2 | 1 |
|  | 2 | 2 | 2 | 2 | 4 | -99 | 49.8 | 3 | 3 | 3 | 1 | 1 | 2 | 1 |
|  | 2 | 2 | 2 | 2 | 4 | -99 | 49.8 | 3 | 3 | 3 | 1 | 2 | 2 | 1 |
|  | 2 | 2 | 2 | 2 | 4 | -99 | 49.8 | 3 | 3 | 3 | 1 | 2 | 2 | 1 |
|  | 2 | 2 | 2 | 2 | 4 | -99 | 49.8 | 3 | 3 | 3 | 1 | 1 | 2 | 1 |
|  | 2 | 2 | 2 | 2 | 4 | -99 | 49.8 | 3 | 3 | 3 | 1 | 1 | 2 | 1 |
|  | 2 | 2 | 2 | 2 | 4 | -99 | 49.8 | 3 | 3 | 3 | 1 | 2 | 2 | 1 |
|  | 2 | 2 | 2 | 2 | 4 | -99 | 49.8 | 3 | 3 | 3 | 1 | 2 | 2 | 1 |
|  | 2 | 2 | 2 | 2 | 4 | -99 | 49.8 | 3 | 3 | 3 | 1 | 1 | 2 | 1 |
|  | 2 | 2 | 2 | 2 | 4 | -99 | 49.8 | 3 | 3 | 3 | 1 | 1 | 2 | 1 |
|  | 2 | 2 | 2 | 2 | 4 | -99 | 49.8 | 3 | 3 | 3 | 1 | 1 | 2 | 1 |
|  | 2 | 2 | 2 | 2 | 4 | -99 | 49.8 | 3 | 3 | 3 | 1 | 1 | 2 | 1 |

**Table S3 continued**

| **Authors** | **[A]** | **[B]** | **[C]** | **[D]** | **[E]** | **[F]** | **[G]** | **[H]** | **[I]** | **[J]** | **[K]** | **[L]** | **[M]** | **[N]** |
| --- | --- | --- | --- | --- | --- | --- | --- | --- | --- | --- | --- | --- | --- | --- |
| Hakvoort et al. (2010) | 1 | 1 | 1 | 2 | 3 | -99 | 42 | 2 | 2 | 1 | 1 | 1 | 2 | 1 |
|  | 1 | 1 | 2 | 2 | 3 | -99 | 42 | 2 | 2 | 1 | 1 | 1 | 2 | 1 |
| Hindman et al. (2013) | 1 | 1 | 1 | 2 | 3 | -99 | 56 | 3 | 3 | 1 | 1 | 1 | 2 | 1 |
|  | 1 | 1 | 1 | 2 | 3 | -99 | 56 | 3 | 3 | 1 | 1 | 1 | 2 | 1 |
|  | 1 | 1 | 1 | 2 | 3 | -99 | 56 | 3 | 3 | 1 | 1 | 1 | 2 | 1 |
|  | 1 | 1 | 1 | 2 | 3 | -99 | 56 | 3 | 3 | 1 | 1 | 1 | 2 | 1 |
|  | 1 | 1 | 1 | 2 | 3 | -99 | 56 | 3 | 3 | 1 | 1 | 1 | 2 | 1 |
|  | 1 | 1 | 1 | 2 | 3 | -99 | 56 | 3 | 3 | 1 | 1 | 1 | 2 | 1 |
|  | 1 | 1 | 1 | 2 | 3 | -99 | 56 | 3 | 3 | 1 | 1 | 1 | 2 | 1 |
|  | 1 | 1 | 1 | 2 | 3 | -99 | 56 | 3 | 3 | 1 | 1 | 1 | 2 | 1 |
|  | 1 | 1 | 1 | 2 | 3 | -99 | 56 | 3 | 3 | 1 | 1 | 1 | 2 | 1 |
|  | 1 | 1 | 1 | 2 | 3 | -99 | 56 | 3 | 3 | 1 | 1 | 1 | 2 | 1 |
|  | 1 | 1 | 1 | 2 | 3 | -99 | 56 | 3 | 3 | 1 | 1 | 1 | 2 | 1 |
|  | 1 | 1 | 1 | 2 | 3 | -99 | 56 | 3 | 3 | 1 | 1 | 1 | 2 | 1 |
|  | 1 | 1 | 1 | 2 | 3 | -99 | 56 | 3 | 3 | 1 | 1 | 1 | 2 | 1 |
|  | 1 | 1 | 1 | 2 | 3 | -99 | 56 | 3 | 3 | 1 | 1 | 1 | 2 | 1 |
| Ingoldsby et al. (2001) | 2 | 1 | 2 | 3 | 1 | -99 | 100 | 3 | 3 | 3 | 2 | 2 | 2 | 1 |
| Iturralde et al. (2013) | 2 | 2 | 1 | 3 | 3 | 2.47 | 40.4 | 2 | 1 | 1 | 1 | 1 | 2 | 1 |
|  | 2 | 2 | 2 | 3 | 3 | 2.47 | 40.4 | 2 | 1 | 1 | 1 | 1 | 2 | 1 |
| Lauretti (2001) | 1 | 1;3 | 1 | 3 | 1 | -99 | 60 | 2 | 1 | 1 | 1 | 1 | 2 | 4 |
|  | 1 | 1;3 | 2 | 3 | 1 | -99 | 60 | 2 | 1 | 1 | 1 | 1 | 2 | 4 |
|  | 1 | 1;3 | 1 | 3 | 1 | -99 | 60 | 2 | 1 | 1 | 1 | 1 | 2 | 4 |
|  | 1 | 1;3 | 2 | 3 | 1 | -99 | 60 | 2 | 1 | 1 | 1 | 1 | 2 | 4 |
| Lindsey et al. (2006) | 2 | 1 | 1 | 2 | 3 | -99 | 100 | 2 | 3 | 1 | 1 | 2 | 2 | 1 |
|  | 2 | 1 | 1 | 2 | 3 | -99 | 100 | 2 | 3 | 2 | 1 | 2 | 2 | 1 |

**Table S3 continued**

| **Authors** | **[A]** | **[B]** | **[C]** | **[D]** | **[E]** | **[F]** | **[G]** | **[H]** | **[I]** | **[J]** | **[K]** | **[L]** | **[M]** | **[N]** |
| --- | --- | --- | --- | --- | --- | --- | --- | --- | --- | --- | --- | --- | --- | --- |
| Liu (2006) | 2 | 1 | 2 | 4 | 3 | 2.66 | -99 | 2 | 3 | 1 | 1 | 1 | 2 | 1 |
|  | 2 | 1 | 2 | 4 | 3 | 2.66 | -99 | 2 | 3 | 1 | 1 | 1 | 2 | 1 |
|  | 1 | 2 | 2 | 4 | 3 | 2.66 | -99 | 2 | 1 | 1 | 1 | 1 | 2 | 1 |
| MacKinnon (1989) | 1 | 1 | 1 | 1 | 2 | -99 | 50 | 2 | 3 | 2 | 1 | 1 | 2 | 1 |
|  | 1 | 1 | 1 | 3 | 2 | -99 | 50 | 2 | 3 | 2 | 1 | 1 | 2 | 1 |
|  | 1 | 1 | 2 | 3 | 2 | -99 | 50 | 2 | 3 | 2 | 1 | 1 | 2 | 1 |
|  | 1 | 1 | 1 | 1 | 2 | -99 | 50 | 2 | 3 | 1 | 1 | 1 | 2 | 1 |
|  | 1 | 1 | 1 | 3 | 2 | -99 | 50 | 2 | 3 | 1 | 1 | 1 | 2 | 1 |
|  | 1 | 1 | 2 | 3 | 2 | -99 | 50 | 2 | 3 | 1 | 1 | 1 | 2 | 1 |
| Masarik & Rogers (2019) | 2 | 3 | 1 | 2 | 3 | -99 | 45.3 | 2 | 3 | 1 | 1 | 1 | 2 | 1 |
|  | 2 | 3 | 1 | 2 | 3 | -99 | 0 | 1 | 3 | 1 | 1 | 1 | 2 | 1 |
|  | 2 | 3 | 1 | 2 | 3 | -99 | 100 | 1 | 3 | 1 | 1 | 1 | 2 | 1 |
|  | 2 | 3 | 1 | 2 | 3 | -99 | 45.3 | 2 | 3 | 1 | 1 | 1 | 2 | 1 |
|  | 2 | 3 | 1 | 2 | 3 | -99 | 45.3 | 2 | 3 | 1 | 1 | 1 | 2 | 1 |
| McGuire et al. (1996) | 1 | 1 | 1 | 2 | 3 | -99 | 42 | 2 | 2 | 1 | 1 | 1 | 2 | 1 |
|  | 1 | 1 | 1 | 2 | 3 | -99 | 42 | 2 | 2 | 1 | 1 | 1 | 2 | 1 |
| McLean (2006) | 2 | 2 | 1 | 2 | 4 | -99 | 41.1 | 3 | 3 | 3 | 1 | 1 | 2 | 4 |
|  | 2 | 2 | 2 | 2 | 4 | -99 | 41.1 | 3 | 3 | 3 | 1 | 1 | 2 | 4 |
| Miller et al. (2012) | 2 | 1 | 2 | 1 | 2 | 1.67 | 49.3 | 2 | 3 | 3 | 2 | 1 | 2 | 1 |

**Table S3 continued**

| **Authors** | **[A]** | **[B]** | **[C]** | **[D]** | **[E]** | **[F]** | **[G]** | **[H]** | **[I]** | **[J]** | **[K]** | **[L]** | **[M]** | **[N]** |
| --- | --- | --- | --- | --- | --- | --- | --- | --- | --- | --- | --- | --- | --- | --- |
| Nagel (1996) | 1 | 1 | 1 | 2 | 3 | -99 | 54.9 | 2 | 1 | 1 | 1 | 1 | 2 | 4 |
|  | 1 | 1 | 2 | 2 | 3 | -99 | 54.9 | 2 | 1 | 1 | 1 | 1 | 2 | 4 |
|  | 1 | 1 | 2 | 3 | 3 | -99 | 54.9 | 2 | 1 | 1 | 1 | 1 | 2 | 4 |
|  | 1 | 1 | 1 | 3 | 3 | -99 | 54.9 | 2 | 1 | 1 | 1 | 1 | 2 | 4 |
|  | 2 | 1 | 1 | 2 | 3 | -99 | 54.9 | 2 | 1 | 1 | 1 | 1 | 2 | 4 |
|  | 2 | 1 | 2 | 2 | 3 | -99 | 54.9 | 2 | 1 | 1 | 1 | 1 | 2 | 4 |
|  | 2 | 1 | 2 | 3 | 3 | -99 | 54.9 | 2 | 1 | 1 | 1 | 1 | 2 | 4 |
|  | 2 | 1 | 1 | 3 | 3 | -99 | 54.9 | 2 | 1 | 1 | 1 | 1 | 2 | 4 |
|  | 2 | 1 | 1 | 2 | 3 | -99 | 54.9 | 2 | 1 | 1 | 1 | 1 | 2 | 4 |
|  | 2 | 1 | 2 | 2 | 3 | -99 | 54.9 | 2 | 1 | 1 | 1 | 1 | 2 | 4 |
|  | 2 | 1 | 2 | 3 | 3 | -99 | 54.9 | 2 | 1 | 1 | 1 | 1 | 2 | 4 |
|  | 2 | 1 | 1 | 3 | 3 | -99 | 54.9 | 2 | 1 | 1 | 1 | 1 | 2 | 4 |
|  | 1 | 3 | 1 | 2 | 3 | -99 | 54.9 | 2 | 1 | 1 | 1 | 1 | 2 | 4 |
|  | 1 | 3 | 2 | 2 | 3 | -99 | 54.9 | 2 | 1 | 1 | 1 | 1 | 2 | 4 |
|  | 1 | 3 | 2 | 3 | 3 | -99 | 54.9 | 2 | 1 | 1 | 1 | 1 | 2 | 4 |
|  | 1 | 3 | 1 | 3 | 3 | -99 | 54.9 | 2 | 1 | 1 | 1 | 1 | 2 | 4 |
|  | 2 | 2 | 1 | 2 | 3 | -99 | 54.9 | 2 | 1 | 1 | 1 | 1 | 2 | 4 |
|  | 2 | 2 | 2 | 2 | 3 | -99 | 54.9 | 2 | 1 | 1 | 1 | 1 | 2 | 4 |
|  | 2 | 2 | 2 | 3 | 3 | -99 | 54.9 | 2 | 1 | 1 | 1 | 1 | 2 | 4 |
|  | 2 | 2 | 1 | 3 | 3 | -99 | 54.9 | 2 | 1 | 1 | 1 | 1 | 2 | 4 |

**Table S3 continued**

| **Authors** | **[A]** | **[B]** | **[C]** | **[D]** | **[E]** | **[F]** | **[G]** | **[H]** | **[I]** | **[J]** | **[K]** | **[L]** | **[M]** | **[N]** |
| --- | --- | --- | --- | --- | --- | --- | --- | --- | --- | --- | --- | --- | --- | --- |
| Odudu (2018) | 1 | 1 | 1 | 3 | 3 | -99 | 45 | 2 | 1 | 1 | 1 | 1 | 2 | 4 |
|  | 1 | 1 | 2 | 3 | 3 | -99 | 45 | 2 | 1 | 1 | 1 | 1 | 2 | 4 |
|  | 2 | 1 | 1 | 3 | 3 | -99 | 45 | 2 | 1 | 1 | 1 | 1 | 2 | 4 |
|  | 2 | 1 | 2 | 3 | 3 | -99 | 45 | 2 | 1 | 1 | 1 | 1 | 2 | 4 |
|  | 1 | 1 | 1 | 2 | 3 | -99 | 45 | 2 | 1 | 1 | 1 | 1 | 2 | 4 |
|  | 1 | 1 | 1 | 2 | 3 | -99 | 45 | 2 | 1 | 1 | 1 | 2 | 2 | 4 |
|  | 1 | 1 | 2 | 2 | 3 | -99 | 45 | 2 | 1 | 1 | 1 | 1 | 2 | 4 |
|  | 1 | 1 | 2 | 2 | 3 | -99 | 45 | 2 | 1 | 1 | 1 | 2 | 2 | 4 |
|  | 2 | 1 | 1 | 2 | 3 | -99 | 45 | 2 | 1 | 1 | 1 | 1 | 2 | 4 |
|  | 2 | 1 | 1 | 2 | 3 | -99 | 45 | 2 | 1 | 1 | 1 | 2 | 2 | 4 |
|  | 2 | 1 | 2 | 2 | 3 | -99 | 45 | 2 | 1 | 1 | 1 | 1 | 2 | 4 |
|  | 2 | 1 | 2 | 2 | 3 | -99 | 45 | 2 | 1 | 1 | 1 | 2 | 2 | 4 |
| Piotrowski et al. (2017) | 2 | 1 | 2 | 1 | 3 | 2.80 | 57.4 | 2 | 1 | 2 | 2 | 1 | 2 | 1 |
|  | 2 | 2 | 2 | 1 | 3 | 2.80 | 57.4 | 2 | 1 | 2 | 2 | 1 | 2 | 1 |
| Query (2000) | 1 | 1 | 1 | 1 | 3 | 2.50 | -99 | 2 | 1 | 1 | 1 | 1 | 2 | 4 |
|  | 1 | 1 | 2 | 1 | 3 | 2.50 | -99 | 2 | 1 | 1 | 1 | 1 | 2 | 4 |
|  | 1 | 1 | 2 | 1 | 3 | 2.50 | -99 | 2 | 1 | 1 | 1 | 1 | 2 | 4 |
|  | 1 | 1 | 1 | 1 | 3 | 2.50 | -99 | 2 | 1 | 1 | 1 | 1 | 2 | 4 |
|  | 1 | 1 | 2 | 1 | 3 | 2.50 | -99 | 2 | 1 | 1 | 1 | 1 | 2 | 4 |
|  | 1 | 1 | 2 | 1 | 3 | 2.50 | -99 | 2 | 1 | 1 | 1 | 1 | 2 | 4 |
| Reese-Weber (2000) | 2 | 2 | 2 | 2 | 4 | 2.80 | 37.1 | 3 | 2 | 1 | 1 | 1 | 2 | 1 |
|  | 1 | 2 | 1 | 2 | 4 | 2.80 | 37.1 | 3 | 2 | 1 | 1 | 1 | 2 | 1 |
| Rinaldi & Howe (2003) | 2 | 1 | 2 | 2 | 3 | 1.20 | 52 | 2 | 3 | 1 | 1 | 1 | 2 | 1 |
|  | 2 | 1 | 2 | 2 | 3 | 1.20 | 52 | 2 | 3 | 1 | 1 | 1 | 2 | 1 |
|  | 2 | 1 | 2 | 2 | 3 | 1.20 | 52 | 2 | 3 | 1 | 1 | 1 | 2 | 1 |
|  | 2 | 1 | 2 | 2 | 3 | 1.20 | 52 | 2 | 3 | 1 | 1 | 1 | 2 | 1 |

**Table S3 continued**

| **Authors** | **[A]** | **[B]** | **[C]** | **[D]** | **[E]** | **[F]** | **[G]** | **[H]** | **[I]** | **[J]** | **[K]** | **[L]** | **[M]** | **[N]** |
| --- | --- | --- | --- | --- | --- | --- | --- | --- | --- | --- | --- | --- | --- | --- |
| Ruff (2012) | 2 | 1 | 1 | 2 | 4 | 2.98 | 51 | 2 | 3 | 1 | 1 | 1 | 2 | 1 |
|  | 2 | 1 | 2 | 2 | 4 | 2.98 | 51 | 2 | 3 | 1 | 1 | 1 | 2 | 1 |
|  | 2 | 1 | 1 | 2 | 4 | 2.98 | 51 | 2 | 3 | 1 | 1 | 2 | 2 | 1 |
|  | 2 | 1 | 2 | 2 | 4 | 2.98 | 51 | 2 | 3 | 1 | 1 | 2 | 2 | 1 |
|  | 2 | 1 | 1 | 2 | 4 | 2.98 | 51 | 2 | 3 | 1 | 1 | 2 | 2 | 1 |
|  | 2 | 1 | 2 | 2 | 4 | 2.98 | 51 | 2 | 3 | 1 | 1 | 2 | 2 | 1 |
| Ruff et al. (2018) | 2 | 1 | 1 | 2 | 3 | 2.94 | -99 | 2 | 3 | 3 | 1 | 1 | 2 | 1 |
|  | 2 | 1 | 1 | 2 | 4 | 2.94 | -99 | 2 | 3 | 3 | 1 | 2 | 2 | 1 |
|  | 2 | 1 | 1 | 2 | 4 | 2.94 | -99 | 2 | 3 | 3 | 1 | 2 | 2 | 1 |
|  | 2 | 1 | 2 | 2 | 3 | 2.94 | -99 | 2 | 3 | 3 | 1 | 1 | 2 | 1 |
|  | 2 | 1 | 2 | 2 | 4 | 2.94 | -99 | 2 | 3 | 3 | 1 | 2 | 2 | 1 |
|  | 2 | 1 | 2 | 2 | 4 | 2.94 | -99 | 2 | 3 | 3 | 1 | 2 | 2 | 1 |
|  | 2 | 1 | 1 | 2 | 3 | 2.94 | -99 | 2 | 3 | 3 | 1 | 1 | 2 | 1 |
|  | 2 | 1 | 1 | 2 | 4 | 2.94 | -99 | 2 | 3 | 3 | 1 | 2 | 2 | 1 |
|  | 2 | 1 | 1 | 2 | 4 | 2.94 | -99 | 2 | 3 | 3 | 1 | 2 | 2 | 1 |
|  | 2 | 1 | 2 | 2 | 3 | 2.94 | -99 | 2 | 3 | 3 | 1 | 1 | 2 | 1 |
|  | 2 | 1 | 2 | 2 | 4 | 2.94 | -99 | 2 | 3 | 3 | 1 | 2 | 2 | 1 |
|  | 2 | 1 | 2 | 2 | 4 | 2.94 | -99 | 2 | 3 | 3 | 1 | 2 | 2 | 1 |
| Scrimgeour (2015) | 1 | 1 | 1 | 1 | 1 | 2.08 | 53 | 2 | 3 | 1 | 1 | 1 | 2 | 4 |
|  | 1 | 1 | 1 | 3 | 1 | 2.08 | 53 | 2 | 3 | 1 | 1 | 1 | 2 | 4 |
|  | 1 | 1 | 1 | 3 | 1 | 2.08 | 53 | 2 | 3 | 1 | 1 | 1 | 2 | 4 |
|  | 1 | 1 | 1 | 1 | 1 | 2.08 | 53 | 2 | 3 | 1 | 1 | 1 | 2 | 4 |
|  | 1 | 1 | 1 | 3 | 1 | 2.08 | 53 | 2 | 3 | 1 | 1 | 1 | 2 | 4 |
|  | 1 | 1 | 1 | 3 | 1 | 2.08 | 53 | 2 | 3 | 1 | 1 | 1 | 2 | 4 |
| Senguttuvan (2014) | 1 | 1 | 1 | 2 | 4 | -99 | 47 | 3 | 3 | 3 | 1 | 1 | 2 | 4 |
|  | 1 | 1 | 2 | 2 | 4 | -99 | 47 | 3 | 3 | 3 | 1 | 1 | 2 | 4 |
|  | 2 | 1 | 1 | 2 | 4 | -99 | 47 | 3 | 3 | 3 | 1 | 1 | 2 | 4 |
|  | 2 | 1 | 2 | 2 | 4 | -99 | 47 | 3 | 3 | 3 | 1 | 1 | 2 | 4 |

**Table S3 continued**

| **Authors** | **[A]** | **[B]** | **[C]** | **[D]** | **[E]** | **[F]** | **[G]** | **[H]** | **[I]** | **[J]** | **[K]** | **[L]** | **[M]** | **[N]** |
| --- | --- | --- | --- | --- | --- | --- | --- | --- | --- | --- | --- | --- | --- | --- |
| Sigda (1999) | 2 | 2 | 1 | 2 | 3 | 3.7 | 37.1 | 2 | 1 | 3 | 2 | 1 | 2 | 4 |
|  | 2 | 2 | 2 | 2 | 3 | 3.7 | 37.1 | 2 | 1 | 3 | 2 | 1 | 2 | 4 |
| Soliday (1996) | 1 | 1 | 1 | 3 | 1 | 3.9 | 48 | 3 | 2 | 1 | 1 | 2 | 2 | 4 |
|  | 1 | 1 | 2 | 3 | 1 | 3.9 | 48 | 3 | 2 | 1 | 1 | 2 | 2 | 4 |
|  | 1 | 1 | 1 | 3 | 1 | 3.9 | 48 | 3 | 2 | 1 | 1 | 2 | 2 | 4 |
|  | 1 | 1 | 2 | 3 | 1 | 3.9 | 48 | 3 | 2 | 1 | 1 | 2 | 2 | 4 |
| Stocker et al. (1997) | 1 | 1 | 1 | 2 | 3 | 3.25 | 45 | 3 | 3 | 1 | 1 | 1 | 2 | 1 |
|  | 1 | 1 | 2 | 2 | 3 | 3.25 | 45 | 3 | 3 | 1 | 1 | 1 | 2 | 1 |
|  | 1 | 1 | 2 | 2 | 3 | 3.25 | 45 | 3 | 3 | 1 | 1 | 1 | 2 | 1 |
|  | 1 | 1 | 1 | 2 | 3 | 3.25 | 45 | 3 | 3 | 1 | 1 | 1 | 2 | 1 |
|  | 1 | 1 | 2 | 2 | 3 | 3.25 | 45 | 3 | 3 | 1 | 1 | 1 | 2 | 1 |
|  | 1 | 1 | 2 | 2 | 3 | 3.25 | 45 | 3 | 3 | 1 | 1 | 1 | 2 | 1 |
| Stocker & Youngblade (1999) | 2 | 1;3 | 1 | 2 | 3 | -99 | 56.6 | 2 | 3 | 3 | 1 | 1 | 2 | 1 |
|  | 2 | 1;3 | 2 | 2 | 3 | -99 | 56.6 | 2 | 3 | 3 | 1 | 1 | 2 | 1 |
|  | 2 | 1;3 | 2 | 2 | 3 | -99 | 56.6 | 2 | 3 | 3 | 1 | 1 | 2 | 1 |
| Tucker et al. (2014) | 2 | 1 | 2 | 1 | 2 | -99 | 51 | 3 | 3 | 3 | 1 | 1 | 2 | 1 |
|  | 2 | 1 | 2 | 1 | 2 | -99 | 51 | 3 | 3 | 3 | 1 | 1 | 2 | 1 |
| Tucker et al. (2020) | 2 | 2 | 2 | 2 | 3 | -99 | 51 | 2 | 3 | 3 | 1 | 1 | 2 | 1 |

**Table S3 continued**

| **Authors** | **[A]** | **[B]** | **[C]** | **[D]** | **[E]** | **[F]** | **[G]** | **[H]** | **[I]** | **[J]** | **[K]** | **[L]** | **[M]** | **[N]** |
| --- | --- | --- | --- | --- | --- | --- | --- | --- | --- | --- | --- | --- | --- | --- |
| Volling et al. (2002) | 1 | 1 | 1 | 1 | 2 | 2.92 | 45 | 2 | 2 | 1 | 1 | 1 | 2 | 1 |
|  | 1 | 1 | 2 | 1 | 2 | 2.92 | 45 | 2 | 2 | 1 | 1 | 1 | 2 | 1 |
|  | 1 | 1 | 1 | 1 | 1 | 2.92 | 45 | 2 | 1 | 1 | 1 | 1 | 2 | 1 |
|  | 1 | 1 | 2 | 1 | 1 | 2.92 | 45 | 2 | 1 | 1 | 1 | 1 | 2 | 1 |
|  | 1 | 1 | 1 | 1 | 2 | 2.92 | 45 | 2 | 2 | 1 | 1 | 1 | 2 | 1 |
|  | 1 | 1 | 2 | 1 | 2 | 2.92 | 45 | 2 | 2 | 1 | 1 | 1 | 2 | 1 |
|  | 1 | 1 | 1 | 1 | 1 | 2.92 | 45 | 2 | 1 | 1 | 1 | 1 | 2 | 1 |
|  | 1 | 1 | 2 | 1 | 1 | 2.92 | 45 | 2 | 1 | 1 | 1 | 1 | 2 | 1 |
|  | 2 | 1 | 1 | 1 | 2 | 2.92 | 45 | 2 | 2 | 1 | 1 | 1 | 2 | 1 |
|  | 2 | 1 | 2 | 1 | 2 | 2.92 | 45 | 2 | 2 | 1 | 1 | 1 | 2 | 1 |
|  | 2 | 1 | 1 | 1 | 1 | 2.92 | 45 | 2 | 1 | 1 | 1 | 1 | 2 | 1 |
|  | 2 | 1 | 2 | 1 | 1 | 2.92 | 45 | 2 | 1 | 1 | 1 | 1 | 2 | 1 |
|  | 2 | 1 | 1 | 1 | 2 | 2.92 | 45 | 2 | 2 | 1 | 1 | 1 | 2 | 1 |
|  | 2 | 1 | 2 | 1 | 2 | 2.92 | 45 | 2 | 2 | 1 | 1 | 1 | 2 | 1 |
|  | 2 | 1 | 1 | 1 | 1 | 2.92 | 45 | 2 | 1 | 1 | 1 | 1 | 2 | 1 |
|  | 2 | 1 | 2 | 1 | 1 | 2.92 | 45 | 2 | 1 | 1 | 1 | 1 | 2 | 1 |
| Weaver-Graham (1998) | 1 | 1 | 1 | 1 | 3 | -99 | 31.4 | 3 | 3 | 3 | 3 | 1 | 2 | 4 |
|  | 1 | 1 | 1 | 1 | 3 | -99 | 46.2 | 3 | 3 | 3 | 2 | 1 | 2 | 4 |
|  | 1 | 1 | 1 | 1 | 3 | -99 | 42.5 | 3 | 3 | 3 | 1 | 1 | 2 | 4 |
| Yu & Gamble (2008) | 1 | 1 | 1 | 1 | 2 | 2.92 | 47 | 2 | 3 | 1 | 1 | 1 | 2 | 1 |
|  | 1 | 1 | 2 | 1 | 2 | 2.92 | 47 | 2 | 3 | 1 | 1 | 1 | 2 | 1 |
|  | 1 | 1 | 2 | 1 | 2 | 2.92 | 47 | 2 | 3 | 1 | 1 | 1 | 2 | 1 |

*Note*. Moderators [A] to [N] are coded according to our coding scheme listed in Table 1. [A] = Operational definition of the interparental relationship ; [B] = Rater of the interparental relationship; [C] = Operational definition of the sibling relationship; [D] = Rater of the sibling relationship; [E] = Mean age of index children; [F] = Mean age difference between siblings (in years); [G] = Gender of index child (percentage male in child sample); [H] = Gender composition of sibling dyad; [I] = Sibling order of index child; [J] = Family type; [K] = Sample type; [L] = Type of study design; [M] = Level of statistical analysis; [N] = Source of publication.
