# Supplementary material for: A systematic review and meta-analysis of the associations between interparental and sibling relationships: Positive or negative?
Source: PLoS One. 2021 Sep 28;16(9):e0257874. doi: 10.1371/journal.pone.0257874 (PMC8478168; doi:10.1371/journal.pone.0257874)
Supplement: S4 Table — (DOCX) [file pone.0257874.s005.docx]

**S4 Table**

*Quality assessment of included studies (k = 47)*

| **Authors** | **Category^a^ for Study Design** | **Quality Criteria^b^** | | | | | | | | | | | | **Comments** |
| --- | --- | --- | --- | --- | --- | --- | --- | --- | --- | --- | --- | --- | --- | --- |
|  |  | **S1** | **S2** | | **1** | | **2** | | **3** | | **4** | **5** | |  |
| McGuire et al. (1996) | 4 | + | | + | | + | + | + | | ? | | | + | S1: goals of the study are given; S2: data/results allowed to answer the research questions; 1: families were participating in a larger longitudinal study of family relationships; 2: clear description of sample (and target population), sample represents the target population; 3: measurements are clearly explained, appropriate variables & measurements for the research questions, internal consistencies given & high; 4: not mentioned how missing data was handled, nonresponse rate not mentioned ; 5: statistical analyses are clearly stated and justified, statistical analyses allowed to answer the research questions |
| Davies et al. (2018) | 4 | + | | + | | + | + | + | | + | | | + | S1: goals/hypotheses of the study are given; S2: data/results allowed to answer the research questions; 1: data came from a longitudinal project on family relationship processes and adolescent development (recruited through local school districts and community centers); 2: clear description of sample (and target population), inclusionary criteria are given, sample overall represents the target population (sample: dominantly white families from middle class backgrounds); 3: measurements are clearly explained, appropriate variables & measurements for the research questions, trained coders and experimenters, interrater reliability/internal consistencies are given (high), validity is given; 4: amount of missing data: 6 %, FIML was used to retain the full sample for analyses; 5: statistical analyses are clearly stated and justified, statistical analyses allowed to answer the research questions |
| Hindman et al. (2013) | 4 | + | | + | | + | + | + | | + | | | + | S1: hypothesis of the study are given; S2: data/results allowed to answer the research questions; 1: study was part of a larger research project; 2: clear description of sample and target population (middle-class US families), inclusionary criteria are given, sample represents the target population; 3: measurements are clearly explained, appropriate variables & measurements for the research questions, DAS commonly used, internal consistencies (test-retest reliabilities and validity) given & high; 4: data checked for missing data, outliers, and normality; five cases with incomplete data removed, small number of outliers – fell within expected ranges (represent accurate values); 5: statistical analyses are clearly stated and justified, statistical analyses allowed to answer the research questions |
| Brody et al. (1994) | 4 | + | | + | | + | + | + | | ? | | | + | S1: hypothesis/purposes of the study are given; S2: data/results allowed to answer the research questions; 1: subsample of 109 families who participated in an earlier study; 2: clear description of sample and (target population), sample represents the target population; 3: trained coder, measurements are clearly explained, appropriate variables & measurements for the research questions, reliability, internal consistency, validity given & overall high; 4: not mentioned how missing data was handled, nonresponse rate not mentioned (indicated how much data was included in each analysis) (Time 1 assessments (109; original sample) were compared for siblings who were and were not included in the Time 3 evaluation – none of the comparisons approached statistical significance); 5: statistical analyses are clearly stated and justified, statistical analyses allowed to answer the research questions |
| Volling et al. (2002) | 4 | + | | + | | + | + | + | | + | | | + | S1: research questions of the study are given; S2: data/results allowed to answer the research questions; 1: recruited from birth announcements, local day-care centers and through referrals from participating families; 2: clear description of sample and (target population), inclusionary criteria are given, sample represents the target population; 3: trained coders, measurements are clearly explained, appropriate variables & measurements for the research questions; reliability, internal consistency, validity given & overall high; 4: nonresponse rate given (low), method to be able to include data of the youngest children (did not respond to all vignettes – missing data were not randomly distributed within the sample); 5: statistical analyses are clearly stated and justified, statistical analyses allowed to answer the research questions |
| Tucker et al. (2014) | 4 | + | | + | | + | + | + | | + | | | + | S1: research questions/hypothesis of the study are given; S2: data/results allowed to answer the research questions; 1: random digital dialing (RDD), sample weights; 2: clear description of sample and target population, inclusionary criteria are given, sample represents the target population; 3: measurements are clearly explained, appropriate variables & measurements for the research questions; low to questionable internal consistency, reliability (but discussed); 4: details of the nonresponse analyses can be obtained from the authors; 5: statistical analyses are clearly stated and justified, statistical analyses allowed to answer the research questions |
| Piotrowski et al. (2017) | 4 | + | | + | | + | + | + | | ? | | | + | S1: purposes of the study are given; S2: data/results allowed to answer the research questions; 1: variety of means including newspaper ads, posters, and mail flyers; 2: clear description of sample (and target population), inclusionary criteria are given, sample overall represents the target population (limited in terms ethno-cultural and socioeconomic diversity; small sample size); 3: measurements are clearly explained, appropriate variables & measurements for the research questions, CBCL widely used, interrater reliability/internal consistencies (Cronbach’s ⍺) are given (high) (Violence Exposure Scale for (young) Children: Cronbach’s ⍺ = 0.56); 4: not mentioned how missing data was handled, nonresponse rate not mentioned (indicated how much data was included in each analysis); 5: statistical analyses are clearly stated and justified, statistical analyses allowed to answer the research questions |
| MacKinnon (1989) | 3 | + | | + | | + | + | ? | | + | | | + | S1: purposes/research questions of the study are given; S2: data/results allowed to answer the research questions; 1: clear description of sample (and target population); inclusionary and exclusionary criteria are given; sample represents the target population; 2: trained observers, measurements are clearly explained, appropriate variables & measurements for the research questions, interrater reliability, internal consistencies, test-retest reliability are given (overall high); 3: not mentioned if the outcome data is complete; 4: appropriate methods to control for confounders are used (families were matched for their socioeconomic status; the unit of analysis was standardized); 5: divorced mothers had been separated for at least 1 year – these were first marriages for these mothers, none had remarried, and all had primary physical custody of their children |
| Sigda (1999) | 4 | + | | + | | + | + | + | | ? | | | + | S1: hypotheses of the study are given; S2: data/results allowed to answer the research questions; 1: families were recruited through juvenile court counselors in three court districts in North Carolina; recruitment strategies varied by court district, and were developed jointly by project staff and juvenile court staff; 2: clear description of sample (and target population), inclusionary criteria are given, sample represents the target population; 3: measurements are clearly explained, appropriate variables & measurements for the research questions, CBCL widely used (adequate reliability and validity), SRQ widely used (adequate reliability and internal consistency), internal consistencies and test-retest and interrater reliabilities are given and moderate to high; 4: not mentioned how missing data was handled, nonresponse rate not mentioned; 5: statistical analyses are clearly stated and justified, statistical analyses allowed to answer the research questions |
| Nagel (1996) | 4 | + | | + | | + | + | + | | ? | | | + | S1: hypotheses of the study are given; S2: data/results allowed to answer the research questions; 1: families were recruited through a wide variety of locations in southern California (public schools, daycare/recreational facilities, …); 2: clear description of sample (and target population), inclusionary criteria are given, sample represents the target population; 3: measurements are clearly explained, appropriate variables & measurements for the research questions, CES-D widely used (good internal consistency), PDS widely used (good reliability and validity); all study measures were tested and revised in an extensive pilot study; interrater and test-retest reliability and internal consistencies are given and overall high; 4: not mentioned how missing data was handled, nonresponse rate not mentioned; 5: statistical analyses are clearly stated and justified, statistical analyses allowed to answer the research questions |
| Weaver-Graham (1998) | 3 | + | | + | | ? | + | ? | | ? | | | + | S1: research questions of the study are given; S2: data/results allowed to answer the research questions; 1: clear description of sample (and target population); inclusionary and exclusionary criteria are given; clinical and convenience sample – we do not know if the participants differed from people who did not choose to participate or from those who were outside the solicitation parameters; 2: measurements and variables are clearly explained, appropriate variables & measurements for the research questions, interrater reliability, internal consistencies, test-retest reliability are given and overall high; 3: not mentioned if the outcome data is complete; 4: children with conduct disorders were not included in the sample so that the issue of other pathology would be less confounding to the findings; regressions were used for analyzing; ‘A matched sample with greater control on demographic variables would help to eliminate the question of confounding variables’; 5: sample from depressed children came from children who were referred for inpatient/outpatient treatment at the WPIC Child and Adolescent Depression Program; children were assessed by a research nurse using the K-SADS-P and K-SADS-E interviews by both parent and child; a psychiatrist interviewed each child and confirmed the diagnosis and each symptom; the high-risk children were required to have at least one first degree and at least one second degree relative with history of childhood onset, recurrent, bipolar or psychotic depression (interviewed) – only children with a depressed parent were included in analysis for the high-risk group; low-risk: families with no first degree relatives with affective or psychotic disorders and no more than 20 % of the second degree relatives could have a lifetime single episode of MDD – children were never diagnosed or treated for psychopathology (K-SADS-E) |
| Lauretti (2001) | 4 | + | | + | | + | + | + | | ? | | | + | S1: aims/research questions of the study are given; S2: data/results allowed to answer the research questions; 1: families contributed to a larger longitudinal study; families recruited through several means (press releases, flyers, parent group meetings at daycare centers, brochures available at many local retail stores); 2: clear description of sample (and target population), inclusionary criteria are given, sample represents the target population; 3: measurements are clearly explained, appropriate variables & measurements for the research questions, internal consistencies and interrater reliability are given and overall acceptable to good; 4: two families were missing some of the relevant data and hence, were excluded from the regression analyses; 5: statistical analyses are clearly stated and justified, statistical analyses allowed to answer the research questions |
| Soliday (1996) | 3 | + | | + | | + | + | + | | - | | | ? | S1: hypotheses/research questions of the study are given; S2: data/results allowed to answer the research questions; 1: clear description of sample (and target population); sample represents the target population (but limited generalizability due to the homogenous sample and the modest sample size); 2: measurements are clearly explained, appropriate variables & measurements for the research questions, internal consistencies, test-retest reliabilities are given and high; 3: 100 % of families who completed Time 1 packets also completed and returned the follow-up; 4: no appropriate methods to control for confounders are used; possible that second-time parents who participated were more motivated in general (rates of participation between first- and second-time parents recruited from parenting groups differed); 5: first-time and second-time parents |
| Lindsey et al. (2006) | 3 | + | | + | | + | + | + | | ? | | | ? | S1: goals/hypothesis of the study are given; S2: data/results allowed to answer the research questions; 1: clear description of sample (and target population), inclusionary criteria are given, sample represents the target population; 2: measurements are clearly explained, appropriate variables & measurements for the research questions, internal consistency given and overall high, validity discussed (exclusively self-report data); 3: Year 1: 247 families, Year 2: 177 families – 11 of the 70 families withdrew from the study, others: children being “lost” at the second assessment (e.g. changed school); analyses comparting the 177 children who participated in all phases of the study with the 69 on whom no classroom data were collected in year 2 revealed no significant differences; 4: regressions were used for analyzing; 5: divorced families: headed by a single mother who had been separated or divorced for more than a year from the boys’ biological fathers |
| Tucker et al. (2019) | 4 | + | | + | | + | + | ? | | + | | | + | S1: research questions/hypothesis of the study are given; S2: data/results allowed to answer the research questions; 1: random digital dialing (RDD), sample weights; 2: clear description of sample and target population, inclusionary criteria are given, sample represents the target population; 3: measurements are clearly explained, appropriate variables & measurements for the research questions; internal consistency, reliability, validity not given or discussed; 4: details of the nonresponse analyses can be obtained from the authors; 5: statistical analyses are clearly stated and justified, statistical analyses allowed to answer the research questions |
| Grych et al. (2004) | 4 | + | | + | | + | + | + | | ? | | | + | S1: goals/hypothesis of the study are given; S2: data/results allowed to answer the research questions; 1: recruited from a large, ethnically diverse public high school in a medium-sized Midwestern city; 2: clear description of sample (and target population), sample represents the target population; 3: measurements are clearly explained, appropriate variables & measurements for the research questions, CPIC (satisfactory levels of internal consistency and test-retest reliability), IPPA (adequate reliability), Youth Self-Report (adequate internal consistency); 4: not mentioned how missing data was handled, nonresponse rate not mentioned; 5: statistical analyses are clearly stated and justified, statistical analyses allowed to answer the research questions |
| Senguttuvan (2014) | 4 | + | | + | | + | + | + | | ? | | | + | S1: research questions/hypothesis of the study are given; S2: data/results allowed to answer the research questions; 1: Study 1 used date from the Purdue Parent Adolescent and Sibling Study (PPASS). Families were sampled from one Midwestern state and the study oversampled ethnic minorities. Study 2 also sampled from one Midwestern state consisted of a nationally representative data set of adolescents with ethnic minorities oversamples as well. 2: clear description of sample (and target population), sample represents the target population; 3: measurements are clearly explained, appropriate variables & measurements for the research questions; 4: Authors compared respondents to non-respondent on key variables and decided to employ pairwise deletion was employed when participants had missing data on outcomes or correlates. 5: statistical analyses are clearly stated and justified, statistical analyses allowed to answer the research questions. |
| Hakvoort et al. (2010) | 4 | + | | + | | + | + | + | | ? | | | + | S1: aims of the study are given; S2: data/results allowed to answer the research questions; 1: three methods: random sample of families who met the criteria from the population register of two cities in the Netherlands; families were contacted through six elementary schools; snowball method; 2: clear description of sample (and target population), inclusionary criteria are given, sample overall represents the target population (more girls than boys); 3: measurements are clearly explained, appropriate variables & measurements for the research questions, reliability is given (Cronbach’s ⍺; questionable to good); 4: not mentioned how missing data was handled, nonresponse rate not mentioned; 5: statistical analyses are clearly stated and justified, statistical analyses allowed to answer the research questions |
| Ruff et al. (2018) | 4 | + | | + | | + | + | + | | ? | | | + | S1: research questions/hypothesis/aims of the study are given; S2: data/results allowed to answer the research questions; 1: sample subset taken from the larger FFP dataset; 2: clear description of sample (and target population), sample overall represents the target population (limited variability in parental conflict and triangulation; relatively affluent and homogeneous participants); 3: measurements are clearly explained, appropriate variables & measurements for the research questions, reliability is given (Cronbach’s ⍺; acceptable to good); 4: questionnaires were screened for missing answers and double markings to prevent them; 5: statistical analyses are clearly stated and justified, statistical analyses allowed to answer the research questions; |
| Brody et al. (1987) | 4 | + | | + | | + | + | + | | ? | | | + | S1: research questions of the study are given; S2: data/results allowed to answer the research questions; 1: recruited through direct telephoning; 2: clear description of sample and target population, inclusionary criteria are given, sample represents the target population; 3: trained observers, measurements are clearly explained, appropriate variables & measurements for the research questions, interrater reliability, internal consistency, test-retest reliability and validity given and high; 4: not mentioned how missing data was handled, nonresponse rate not mentioned (one time indicated how much data was included in the analysis (41 of 42)); 5: statistical analyses are clearly stated and justified, statistical analyses allowed to answer the research questions; |
| Button & Gealt (2010) | 4 | + | | + | | + | + | + | | ? | | | + | S1: goals of the study are given; S2: data/results allowed to answer the research questions; 1: data came from a sample of Delaware public school students (Delaware School Survey); 2: clear description of sample (and target population), sample represents the target population; 3: measurements are clearly explained, variables are clearly defined and accurately measured, appropriate variables & measurements, Cronbach’s α is stated (questionable to acceptable); 4: not mentioned how missing data was handled, nonresponse rate not mentioned; 5: statistical analyses are clearly stated, statistical analyses allowed to answer the research questions |
| Ingoldsby et al. (2001) | 4 | + | | + | | + | + | + | | + | | | + | S1: research questions/hypotheses of the study are given; S2: data/results allowed to answer the research questions; 1: recruited from Women, Infant, and Children Nutritional Supplement Program clinics; 2: clear description of sample and target population (low-income boys), inclusionary criteria are given, sample represents the target population; 3: measurements are clearly explained, appropriate variables & measurements for the research questions, CTS (adequate reliability and validity; widely used), STRS (good reliability), SCCS (good reliability); 4: 11 % attrition for the total sample (modest); subjects with missing data not included in the regressions; 5: statistical analyses are clearly stated and justified, statistical analyses allowed to answer the research questions |
| Liu (2006) | 4 | + | | + | | + | + | ? | | ? | | | + | S1: research questions/hypotheses of the study are given; S2: data/results allowed to answer the research questions; 1: A community sample of families was recruited through flyers distributed in schools, libraries, community centers, physician’ offices, as well as through advertisements placed in magazines and newspapers. 2: clear description of sample (and target population), sample represents the target population; 3; The authors have clearly defined all measures and included relevant indicators of the validity/reliability of the tested measures, with the exception of the measure for marital aggression*.* The author has not included any information about the validity/reliability of the *Domestic Conflict Inventory;* 4: Authors do not report any information in relation to nonresponse bias. Since this is a longitudinal study, this would be a particularly important aspect to consider; 5: statistical analyses are clearly stated and justified, statistical analyses allowed to answer the research questions. |
| Stocker & Youngblade (1999) | 4 | + | | + | | + | + | + | | ? | | | + | S1: goals of the study are given; S2: data/results allowed to answer the research questions; 1: recruited from public schools and from advertisements placed in local newspapers; 2: clear description of sample (and target population), sample represents the target population; 3: measurements are clearly explained, appropriate variables & measurements for the research questions; interrater reliability, internal consistency given and overall high; 4: not mentioned how missing data was handled, nonresponse rate not mentioned; 5: statistical analyses are clearly stated, statistical analyses allowed to answer the research questions |
| Stocker et al. (1997) | 4 | + | | + | | + | + | + | | ? | | | + | S1: goals of the study are given; S2: data/results allowed to answer the research questions; 1: recruited from public schools in suburban communities; families were participating in a larger study; 2: clear description of sample (and target population), inclusionary criteria are given, sample represents the target population; 3: measurements are clearly explained, appropriate variables & measurements for the research questions; internal consistency, (validity) given and overall high; 4: not mentioned how missing data was handled, nonresponse rate not mentioned; no differences between the current sample and the larger sample; 5: statistical analyses are clearly stated, statistical analyses allowed to answer the research questions |
| Reese-Weber (2000) | 3 | + | | + | | + | + | ? | | + | | | + | S1: research questions of the study are given; S2: data/results allowed to answer the research questions; 1: clear description of sample (and target population), inclusionary criteria are given, sample represents the target population; 2: measurements are clearly explained, appropriate variables & measurements for the research questions; internal consistency (reliability) given and overall high; 3: not mentioned if the outcome data is complete; 4: the late adolescent sample was all college students (possible confounder); middle adolescents: from relatively middle-class homes (may not have represented the general population of middle adolescents and may have more closely matched the late adolescent college sample); 5: separate criteria and procedures for Middle Adolescent and Late Adolescent samples |
| Dekovic & Buist (2005) | 4 | + | | + | | - | + | + | | ? | | | + | S1: aims of the study are given; S2: data/results allowed to answer the research questions; 1: sampling strategy not mentioned; 2: clear description of sample (and target population), sample represents the target population; 3: trained interviewers, measurements are clearly explained, appropriate variables & measurements for the research questions; internal consistency, reliabilities, (validity) given and overall high; 4: not mentioned how missing data was handled, nonresponse rate not mentioned; 5: statistical analyses are clearly stated, statistical analyses allowed to answer the research questions |
| Erel et al. (1998) | 4 | + | | + | | - | + | + | | ? | | | + | S1: research questions of the study are given; S2: data/results allowed to answer the research questions; 1: sampling strategy not mentioned; 2: clear description of sample (and target population), inclusionary criteria are given, sample represents the target population; 3: trained coders, measurements are clearly explained, appropriate variables & measurements for the research questions; internal consistency, test-retest reliability, validity given and high; 4: not mentioned how missing data was handled, nonresponse rate not mentioned; 5: statistical analyses are clearly stated, statistical analyses allowed to answer the research questions |
| Dawson et al. (2014) | 4 | + | | + | | + | + | + | | ? | | | + | S1: hypothesis/research questions of the study are given; S2: data/results allowed to answer the research questions; 1: recruited by leaflets in schools (97 %) and advertisements in local papers (3 %); 2: clear description of sample (and target population), inclusionary criteria are given, sample represents the target population; 3: measurements are clearly explained, appropriate variables & measurements for the research questions; internal consistency, interrater reliability given and high; 4: not mentioned how missing data was handled, nonresponse rate not mentioned; 5: statistical analyses are clearly stated, statistical analyses allowed to answer the research questions |
| Yu & Gamble (2008) | 4 | + | | + | | + | + | + | | ? | | | + | S1: hypothesis/research questions of the study are given; S2: data/results allowed to answer the research questions; 1: data were drawn from a study of the socialization of children’s emotions; 2: clear description of sample (and target population), inclusionary criteria are given, sample represents the target population; 3: measurements are clearly explained, appropriate variables & measurements for the research questions; internal consistency given and overall high; 4: not mentioned how missing data was handled, nonresponse rate not mentioned (indicated how much data was included in each analysis); 5: statistical analyses are clearly stated, statistical analyses allowed to answer the research questions |
| McLean (2006) | 4 | + | | + | | + | + | + | | ? | | | + | S1: research questions/hypothesis of the study are given; S2: data/results allowed to answer the research questions; 1: Participants were obtained from two Protestant churches, one located in Brooklyn, NY, and one located in Queens, NY. Participants were also obtained by referrals made by friends and family members of the researchers. 2: clear description of sample (and target population), sample represents the target population; 3: measurements are clearly explained, appropriate variables & measurements for the research questions; 4: authors do not report any information in relation to nonresponse bias; 5: statistical analyses are clearly stated and justified, statistical analyses allowed to answer the research questions. |
| Rinaldi & Howe (2003) | 4 | + | | + | | + | + | + | | ? | | | + | S1: purpose/research questions of the study are given; S2: data/results allowed to answer the research questions; 1: families were recruited through local schools; 2: clear description of sample (and target population), inclusionary criteria are given, sample represents the target population; 3: measurements are clearly explained, appropriate variables & measurements for the research questions; internal consistencies given and high; 4: not mentioned how missing data was handled, nonresponse rate not mentioned; 5: statistical analyses are clearly stated, statistical analyses allowed to answer the research questions |
| Iturralde et al. (2013) | 4 | + | | + | | + | + | + | | ? | | | + | S1: goals/hypotheses of the study are given; S2: data/results allowed to answer the research questions; 1: the participating sibling pairs were part of the third wave of a larger longitudinal study; families were recruited from a major metropolitan area through newspaper advertising, flyers, and word-of-mouth; 2: clear description of sample (and target population), inclusionary and exclusionary criteria are given, sample represents the target population; 3: measurements are clearly explained, appropriate variables & measurements for the research questions; CPIC commonly used and well-validated; internal consistencies and interrater reliability given and overall high; validity analysis: coefficients were generally weak to moderate but consistent with expected associations; 4: not mentioned how missing data was handled, nonresponse rate not mentioned; 5: statistical analyses are clearly stated, statistical analyses allowed to answer the research questions |
| Beyers Carlson (2018) | 4 | + | | + | | ? | + | + | | + | | | + | S1: research questions/hypothesis of the study are given; S2: data/results allowed to answer the research questions; 1: the authors mention that the participating families came from a previous longitudinal study and provide a reference to the original investigation; although they do not provide any information in regards to the sample strategy, they do describe how they selected the sample which participated in their study (re-contacting participants to take part in further follow-up assessments); thus the original sampling strategy is not clear; 2: clear description of sample (and target population), sample represents the target population; 3: measurements are clearly explained, appropriate variables & measurements for the research questions; 4: ratings correspond to Study 2 (p. 57-91) only, as this study focuses on aspects of the interparental and sibling relationship quality. 4. Reasons for non-response were discussed (p. 77), responders vs. nonresponders were compared on demographic characteristics (p. 77) and statistical compensation for nonresponse was performed via multiple imputations (p. 85); 5: statistical analyses are clearly stated and justified, statistical analyses allowed to answer the research questions. |
| Ruff (2012) | 4 | + | | + | | + | + | + | | ? | | | + | S1: research questions/hypothesis of the study are given; S2: data/results allowed to answer the research questions; 1: participant families from Seattle, Washington were selected using a purchased national telephone survey database; randomly selected familied from targeted census tracts that mirrored the reported socio-economic and racial stratification of local school districts were used; researchers deemed families eligible if they had a child between the ages of 10-14; 2: clear description of sample (and target population), sample represents the target population; 3: measurements are clearly explained, appropriate variables & measurements for the research questions; 4: while the authors assessed for missing data and report that there were 4-6% missing data, they did not consider whether respondents and non-respondents differed on the variables of interest, nor did they perform any statistical compensation for nonresponse; instead the authors used listwise deletion in their analysis, justifying this by explaining that there was minimal missing data (p. 47); 5: statistical analyses are clearly stated and justified, statistical analyses allowed to answer the research questions. |
| Guinn et al. (2012) | 4 | + | | + | | + | + | + | | + | | | + | S1: research questions/hypothesis of the study are given; S2: data/results allowed to answer the research questions; 1. Families were recruited through flyers at multiple community locations; families who contacted the lab manager and represented two-parent families with at least one child in middle childhood were asked to participate; 2: clear description of sample (and target population), sample represents the target population; 3: measurements are clearly explained, appropriate variables & measurements for the research questions; 4: missing values were deemed to be missing completely at random and estimation maximization and casewise deletion procedures were to used to address missing values; 5: statistical analyses are clearly stated and justified, statistical analyses allowed to answer the research questions. |
| Caya (2001) | 4 | + | | + | | - | + | + | | ? | | | + | S1: research questions/hypothesis of the study are given; S2: data/results allowed to answer the research questions; 1: The authors mention that the participants came from a public school in central  Massachusetts and that the students were recruited through their third-grade classrooms. No information in regards to the sampling strategy is given; 2: clear description of sample (and target population), sample represents the target population; 3: measurements are clearly explained, appropriate variables & measurements for the research questions; 4: not mentioned how missing data was handled, nonresponse rate not mentioned (indicated how much data was included in each analysis) 5: statistical analyses are clearly stated and justified, statistical analyses allowed to answer the research questions. |
| Masarik & Rogers (2019) | 4 | + | | + | | + | + | + | | + | | | + | S1: research questions/hypotheses of the study are given; S2: data/results allowed to answer the research questions; 1: majority of participants were recruited from middle schools in the community, rather than self-selected volunteers from advertising; 2: clear description of sample (and target population), inclusionary criteria are given, sample represents the target population; 3: trained observers/coders, adequate interrater reliability; measurements are clearly explained, appropriate variables & measurements for the research questions; internal consistencies given and high; 4: full information maximum likelihood estimation was used to account for missing data, as estimates have been shown to be less biased and more efficient compared to other forms of estimation; 5: statistical analyses are clearly stated, statistical analyses allowed to answer the research questions |
| Dunn et al. (1999) | 4 | + | | + | | + | + | + | | ? | | | + | S1: research questions of the study are given; S2: data/results allowed to answer the research questions; 1: sample was part of the ALSPAC (Avon Longitudinal Study of Pregnancy and Childhood; included all the women in the Avon Health District ho gave birth between 1 April 1991 and 31 December 1992); 2: clear description of sample (and target population), inclusionary and exclusionary criteria are given, sample represents the target population; 3: measurements are clearly explained, appropriate variables & measurements for the research questions; widely used interview schedule (good agreement with observational measures); validity has been established; internal consistencies given and overall acceptable to good; 4: level of retention of the sample over the 5 years: 75 % (within the range reported for large-scale surveys); missing data at the various time-points mean that the sample was further reduced for the overtime analyses, and the sample size varied according to the variables under scrutiny; 5: statistical analyses are clearly stated, statistical analyses allowed to answer the research questions |
| Miller et al. (2012) | 4 | + | | + | | + | + | + | | + | | | + | S1: hypothesis of the study are given; S2: data/results allowed to answer the research questions; 1: participants were recruited using flyers with assistance from the Head Start Community Action Agency offices in two Michigan counties; study was part of a Head-Start University Partnership Program; 2: clear description of sample (and target population), sample represents the target population; 3: measurements are clearly explained, appropriate variables & measurements for the research questions; internal consistencies given and acceptable to good; test-retest reliability given one time (poor); 4: statistical compensation for nonresponse (multiple imputation); 5: statistical analyses are clearly stated, statistical analyses allowed to answer the research questions |
| Brockman (1994) | 4 | + | | + | | + | + | + | | + | | | + | S1: research questions/hypothesis of the study are given; S2: data/results allowed to answer the research questions; 1: families were recruited through elementary and junior high schools in West Virginia; families who satisfied the screening criteria were selected to participate in the study; 2: clear description of sample (and target population), sample represents the target population; 3: measurements are clearly explained, appropriate variables & measurements for the research questions; 4: z-score comparisons reveled no significant differences in the total proportion of missing data for older and younger adolescents; due to unequal and in many instances unacceptably small cell sized, correlation instead of MANOVA analyses were conducted to examine patterns of nonresponse among older and younger adolescents; missing data values were replaced with the appropriate variable means for each group; 5: statistical analyses are clearly stated and justified, statistical analyses allowed to answer the research questions. |
| Conners (1999) | 4 | + | | + | | + | ? | + | | ? | | | + | S1: research questions/hypothesis of the study are given; S2: data/results allowed to answer the research questions; 1: participants were recruited through middle schools, community groups and churches; 2: The sample consisted of 70 adolescent females. It is unclear why no males were included; 3: measurements are clearly explained, appropriate variables & measurements for the research questions; 4: not mentioned how missing data was handled, nonresponse rate not mentioned (indicated how much data was included in each analysis); 5: statistical analyses are clearly stated and justified, statistical analyses allowed to answer the research questions. |
| Query (2000) | 4 | + | | + | | + | + | + | | ? | | | + | S1: research questions/hypothesis of the study are given; S2: data/results allowed to answer the research questions; 1: two recruitment strategies were employed; the initial one elicited participation through telephone calls; the second one recruited families through print media, including flyers and a newspaper column; 2: clear description of sample (and target population), sample represents the target population; 3: measurements are clearly explained, appropriate variables & measurements for the research questions; 4: not mentioned how missing data was handled, nonresponse rate not mentioned (indicated how much data was included in each analysis); 5: statistical analyses are clearly stated and justified, statistical analyses allowed to answer the research questions. |
| Haj-Yahia & Abdo-Kaloti (2003) | 4 | + | | + | | + | + | + | | ? | | | + | S1: research questions of the study are given; S2: data/results allowed to answer the research questions; 1: sample was drawn from seven secondary schools in the West Bank and six secondary schools in East Jerusalem; three classes were chosen randomly from each school; 2: clear description of sample (and target population), sample represents the target population; 3: measurements are clearly explained, appropriate variables & measurements for the research questions; internal consistencies given and acceptable to good; validity and reliability are mentioned one time; 4: not mentioned how missing data was handled, nonresponse rate not mentioned (indicated how much data was included in each analysis); 5: statistical analyses are clearly stated, statistical analyses allowed to answer the research questions |
| Odudu (2018) | 4 | + | | + | | ? | + | + | | + | | | + | S1: research questions/hypothesis of the study are given; S2: data/results allowed to answer the research questions; 1: No information in regards to the sampling strategy is given; 2: clear description of sample (and target population), sample represents the target population; 3: measurements are clearly explained, appropriate variables & measurements for the research questions; 4: 10% of missing data was found; it was found that data was missing at random; expectation maximization was used to account for missing data by adding estimated values on the data; additionally multiple imputations with 5 iterations were performed to fill in missing data based on the presence of missing values at random; 5: statistical analyses are clearly stated and justified, statistical analyses allowed to answer the research questions. |
| Scrimgeour (2015) | 4 | + | | + | | + | + | + | | + | | | + | S1: research questions/hypothesis of the study are given; S2: data/results allowed to answer the research questions; 1: families were recruited through newspaper birth announcements, flyers posted at local businesses, a university newsletter, and a database of local families interested in participating in research; 2: clear description of sample (and target population), sample represents the target population; 3: measurements are clearly explained, appropriate variables & measurements for the research questions; 4: a max. of 24.3 % of the key variables were missing; father age and younger sibling age were associated with missing data thus these variables were included as covariates in all relevant models; 5: statistical analyses are clearly stated and justified, statistical analyses allowed to answer the research questions. |
| Dantchev & Wolke (2019) | 4 | + | | + | | + | + | + | | + | | | + | S1: aims of the study are given; S2: data/results allowed to answer the research questions; 1: pregnant women from Avon (United Kingdom) with an expected delivery date between April 1, 1991 and December 31, 1992 were recruited; 2: clear description of sample (and target population), inclusionary and exclusionary criteria are given, sample represents the target population; 3: measurements are clearly explained, appropriate variables & measurements for the research questions; internal consistencies given and acceptable; 4: statistical compensation for nonresponse (multiple imputation); 5: statistical analyses are clearly stated, statistical analyses allowed to answer the research questions |

*Note:* Quality of studies were assessed using the Mixed Methods Appraisal Tool (MMAT; Hong et al., 2019)*.* ^a^Study design was assigned to the MMAT design categories: 1 = Qualitative; 2 = Quantitative randomized controlled trials; 3 = Quantitative non-randomized; 4 = Quantitative descriptive; 5 = Mixed methods. ^b^Two general quality criteria (S1 and S2) and five design-specific quality criteria (1 to 5) were assessed: + = criterion is met; - = criterion is not met; ? = criterion is not assessable.
