# Supplementary material for: A systematic review and meta-analysis of the associations between interparental and sibling relationships: Positive or negative?
Source: PLoS One. 2021 Sep 28;16(9):e0257874. doi: 10.1371/journal.pone.0257874 (PMC8478168; doi:10.1371/journal.pone.0257874)
Supplement: Attachment — Submitted filename: Response to Reviewers.pdf [file pone.0257874.s006.pdf]

Dear Editor,
Dear Reviewers,

We would like to thank the editor and the two anonymous reviewers for the helpful comments about our manuscript (formerly entitled “A systematic review and meta-analysis of the associations between interparental and sibling relationships: Spillover or compensation?”) and the time spent providing us feedback. We are very pleased with the reviewers’ overall positive appraisal and appreciate their attention to areas where our work could be strengthened. The reviewers raised important points. We have carefully considered and responded to each point raised by the reviewers and made several changes to the manuscript (see file labeled 'Revised Manuscript with Track Changes'). As requested, we also submit an unmarked version of the manuscript without tracked changes (see file labeled 'Manuscript'). The **page numbers** we provided in this response letter refer to the second, **unmarked version** of the manuscript. Our report follows the PRISMA guidelines (see S1 Appendix for the PRISMA checklist).

To comprehensively respond to all reviewer feedback, we pasted their comments into this document. Our responses are noted with **Author Response** in **bold** type and we note the location of any changes made to the manuscript. In all, we think these revisions resulted in a more thorough, nuanced presentation of the theoretical background and a higher quality piece of work overall.

Once again, thank you very much for the time and energy you devoted to this manuscript. We believe this revision is markedly better after receiving your feedback.

Sincerely,

Authors

Reviewer #1:

This is a very well-written manuscript describing a meta-analysis examining the relation between interparental relationship quality (IPR) and sibling relationship quality (SRQ). Their aims were clear and I liked that they couched the work within a family systems perspective and put two competing hypotheses out there that could, and have been, used to explain the relation between IPR and SRQ: the spillover or compensatory hypothesis. There has been some speculation for years that a positive sibling relationship can buffer the effects of interparental conflict, but I don't believe anyone until now has examined this assumption in a meta-analysis so the information here is timely and could make a contribution to the field. I very much enjoyed reading this paper and found it quite informative.
I have some very minor comments for the authors that might help clarify some of their procedures and decision-making when conducting the meta-analysis.

Author Response: Thank you for this positive appraisal.

Comment 1: Given the central focus on IPR, I think it is important from the start to define or describe what they are including here under the IPR category. It may seem obvious to some, but I wasn't clear what the inclusion criteria were here. For instance, martial relationships clearly would be included, and one of their moderator variables was cohabiting versus non-cohabiting, so there was some consideration of residence. Many would consider the coparenting relationship to be perhaps a central feature of IPR but it appears that the coparenting literature was not examined here or included, so the question I have is why not? Why would the coparenting literature not be included as part of a meta-analysis on IPR? Perhaps there are not many studies out there, which I could see might be the case. But, some justification I believe needs to be included because this was a glaring hole in my read of this paper. So noting up front how they are defining IPR and what is included seems essential.

Author Response: Thank you for bringing this issue to our attention. We agree that it is important to add clarifications about the conceptualization of the IPR used in the present meta-analysis. We exclusively focused on the quality of the IPR, namely the intimate relationship between the parents, and thus excluded studies that only investigated the coparenting alliance. The reasons for excluding the coparenting literature are the following: The concept of the coparenting relationship clearly differs from the IPR. The debate about the empirical independence and interdependence between the IRP and the parents' coparenting alliance has been reflected in the conceptual development of the coparenting construct from the outset (Feinberg, 2003). Coparenting has conceptually been considered as a mediator explaining the association between the IRP and parenting skills (Margolin et al., 2001). Thus, the IRP and coparenting are indeed mutually interdependent, but conceptually distinct constructs. We should therefore not use both constructs in an interchangeable way for our meta-analysis. Hypothesized associations might also differ for IRP and coparenting with regard to their correlation with the sibling relationship. Moreover, indicators to measure coparenting and IRP are not simply comparable. Coparenting is considered a multidimensional construct and relevant theoretical conceptions differ in their postulated dimensions. The most frequently discussed positive coparenting dimensions include cooperation, mutual support, and the division of labor. To the negative coparenting dimensions belong coparenting conflict and undermining. In sum, together with the already complex operationalization of the IPR and sibling relationship, the inclusion of coparenting would pose a particular challenge for a quantitative synthesis of a specific association.

We specified the definition of the interparental relationship in order to distinguish it from the coparenting relationship in the introduction on pages 4/5:

> **"The notion that the quality of the interparental relationship, i.e., the intimate relationship between the parents, is pivotal to children's well-being and that interparental conflict belongs to the strongest predictors of child maladjustment has been established throughout the past several decades [5]."**

Additionally, we specified our inclusion criteria on page 14 concerning this matter:

> **"We exclusively focused on the quality of the interparental relationship, namely the intimate relationship between the parents, and thus excluded studies that only investigated the closely related, but distinct coparenting relationship (i.e., how parents cooperate and coordinate in childrearing and support each other in their parenting efforts)."**

That said, we were very inclusive concerning family types. We included family studies regardless of family type or kinship. That is, biological, adoptive, foster, and step-parents as well as divorced or separated parents were included (cf. moderator [J], page 11). Given that the circumstance whether parents cohabit or are separated may affect the associations between different family relationships, we considered family as moderator. Thus, we examined whether effects differed between (1) cohabiting families (including biological, adoptive, foster, and step-families), (2) non-cohabiting families (including separated and divorced families), and (3) mixed or unknown family types.

<u>Comment 2</u>: Also, on p. 3 when introducing the three aims, perhaps they can just list in parentheses for aim 3, what some of the moderators are that will be included to inform the reader of what is to come. I found myself asking. What moderators?

Author Response: Thank you for this suggestion. Along with a related point from Reviewer #2 concerning clarification of the two major goals, we revised this passage and added specific examples of examined moderators in parentheses on page 3:

> **"First, are the interparental and sibling relationships significantly associated in a positive or negative direction? Second, do different moderators (i. a., operational definition and rater of the relationship quality, child age and sex, age difference between and sex composition of siblings, sibling order, family type, and other study characteristics) affect this association?"**

<u>Comment 3</u>: I understand that certain decisions have to be made when conducting meta-analyses, but I also think there needs to be some note then in the limitations section of the implications of such decisions and how the findings might have differed. I'm thinking specifically of the decision to use data from the younger sibling for the sake of simplicity. I agree that such decisions need to be made, but the literature is also pretty clear that older siblings are more likely to be the leaders, managers, and teachers in sibling relationships, and one might argue protectors or instigators, so perhaps IPR would have a much stronger effect when examining the older siblings than the younger ones. Often times, it is what the older sibling is doing that determines the SRQ. Perhaps the authors would have found stronger effects for compensation if the decision was to extract information on the older sibling versus the younger sibling.

As a follow-up, I was a bit confused later on p. 11 when they claimed they coded sibling order of the index child to include whether they were the younger or youngest sibling or the older or oldest sibling. This seemed to contradict their earlier decision to extract data only from younger siblings. Some clarification here might be helpful.

Author Response: We apologize for the confusion. We did not exclusively focus on the younger sibling. However, for some moderators, we focused on the "index children". The index children were defined as the children who either reported on the sibling relationship quality themselves or for whom the parents or the observer rater reported on the sibling relationship quality. If

characteristics of two (or more) siblings were reported, only data from the younger (or youngest) child were extracted for the sake of simplicity. Additionally, to control for the possibility that the sibling order influences the quality of their relationship, we coded whether the index children, if reported, were the younger/youngest siblings or the elder/eldest siblings (see moderator [I]). We hope that these clarifications are helpful for the reader (see pages 10-11).

Comment 4: I was also not clear on what search terms were actually used. I can understand that a large number of hits could be found using terms such as "child", "maternal", etc. But some information on the iterative process of refinement that was used would be helpful here as I was not clear what "broad terms not specific to dyadic relationship descriptions" were.

Author Response: This is an important point. All search terms we used and an example of a full electronic search query (that is, for Web of Science) are provided in Table S2 as Supporting Information. In the first submission of the manuscript, keywords that were excluded during refinement ("not specific to dyadic relationship descriptions") were listed in brackets, but confusingly labelled as examples. Following your comment, we changed the wording on page 13 to clarify this issue:

> **"(hereby excluded were the terms "child", "mother" or "maternal", and "father" or "paternal")"**

As to the iterative process, we described the introduction of proximity parameters, i.e., operators defining the maximum word distance between two terms in a second step. Iteration consisted of modifying these distance parameters (going up from 0) until only <10% of records in a random subsample were relevant to our study (as suggested by Mikolajewicz and Komarova, see page 13) to ensure broad inclusion. As seen in the example search query in Table S2, this criterion was achieved when allowing up to five words in between terms. We hope these explanations help clarify our search strategy.

Comment 5: P. 13 '…studies were excluded because abstract analysis revealed they were unsuitable for the current review." Again, could the authors simply provide an example or two here of what these situations were that led to exclusion.

Author Response: Thanks for this suggestion. We provided some reasons for exclusions of studies in the abstract analysis on page 13:

> **"Among them, k = 2,593 studies were excluded because abstract analysis revealed that they were unsuitable for the current review (e.g., interparental relationship or sibling relationship not examined, not original empirical research, no quantitative data)."**

Reviewer #2:

This is a very relevant study and has many strengths. The statistical analysis and the study description are very accurate, and authors provided all details and materials to allow study replicability. However, I think that there are major and minor issues that should be addressed. Here are some comments and suggestions that may help authors to further improve their manuscript:

Author Response: We appreciate the acknowledgement of the positive features of our work.

Comment 1: My main concern is about the choice and presentation of the theoretical framework (i.e. framework by Erel and Burman and spillover vs. compensation hypothesis). It seems to me that a more consistent replication of the framework by Erel and Burman about siblings' relationship would be a meta-analysis about the link between parent-child relationship and siblings' relationship, where the feelings experienced by a child in the relation with a parent may spill over in her/his relationship with the sibling. I agree with authors about the presence of a number of reasons to expect that positive interparental relationship quality is associated with positive sibling relationship quality, and vice versa, however not all of them would be explained through the spillover mechanism: spillover mainly refers to an indirect impact on other family members through affect spillover within a person from one family subsystem to another one (i.e. it is an indirect hypothesis about the impact of interparental conflict on child adjustment which is explained trough the deteriorating of parenting practices). Alternative hypotheses to the spillover one, support a direct impact of interparental conflict on child adjustment (e.g. the emotional security hypothesis and the social learning theory), however they are reported in this manuscript in support the spillover mechanism. Also the influence of third family stressors (p. 6 line 130) is reported here as a form of spillover, however it is better known in literature as "crossover effect" (e.g. doi: 10.1037/a0015977 "A third hypothesized process is crossover. Rather than a transfer of affect within one person across subsystems (i.e. spillover), crossover refers to the transfer of affect or behavior between people. An example of crossover is when the stress experienced by one partner at work is detrimental to the other partner's relationship with a child"). I think that these theories refer to different mechanisms (though they are not self-excluding) and should be presented in a more consistent way in the manuscript.

Author Response: Many thanks for these excellent theoretical suggestions. We recognize that our theoretical underpinning in the former manuscript was deficient. The present meta-analysis differentiates from the seminal work by Erel and Burman in that we examined two family relationships where no family member is part of both relationships (i.e., parents and siblings instead of parents and parent-child relations, as it was the case by Erel and Burman). In the original framework, the spillover hypothesis postulates that affect within persons transfers directly from one relationship to another. Thus, it is about the same person in different relationships, which was not investigated in the present meta-analysis. Intuitively, it can be assumed that affect contagion also occurs from one family relationship to the other if no person is involved in both relationships (interparental and sibling relationship, as we examined it). But this is more a potential explanation of our findings and we cannot establish from our data whether interactions in the parent-child relationships mediate our findings. The same applies to the compensation processes, which proposes the transfer of affect within persons between relationships, such that a person will seek satisfaction in one relationship or setting to compensate for shortages in another domain. Hence, it is about the same person in different relationships as well.
Along this line, we revised the entire manuscript: We no longer contrasted spillover and compensation processes, but positive and negative associations between the interparental and the sibling relationship. Spillover and compensation are thereby presented, among others, as possible explanations for these positive or negative associations. The spillover hypotheses, in particular, was introduced in the paragraph about the indirect impact by parenting (see page 5). These major

revisions have particular implications for the title of the article (see page 1), the abstract (see page 2), the introduction (see pages 3-8), and the discussion (see pages 25-28).

Moreover, we thank the Reviewer for the comment on the crossover effect. We specified the relevant passage about the influence of third family stressors and added the mentioned citation, see page 6:

> **"A final factor that could underlie positive associations between the interparental and sibling relationships is the influence of a third family stressor that is neither part of the interparental nor the sibling relationship, such as one parent's stress at work, parental unemployment, or chronic illness of a family member. This process is referred to as crossover, a transfer of affect or behavior between people due to external stressors [23]. In other words, these stressors not only compromise the individual well-being of family members, but also form the basis for a strained family climate, thereby likely triggering conflict in different family relationships [24]."**

<u>Comment 2</u>: I suggest maintaining consistency throughout the paper in the presentation of goals to help the readers (they are presented as 2 or 3 goals in different manuscript's sections).

Author Response: Thank you for bringing this inconsistency to our attention. We aligned the description of the two main study goals throughout the manuscript. Right in the front, on page 3, where there were formerly presented three goals, we revised our wording:

> **"This study aims to examine the following objectives: First, are the interparental and sibling relationships significantly associated in a positive or negative direction? Second, do different moderators (i. a., operational definition and rater of the relationship quality, child age and sex, age difference between and sex composition of siblings, sibling order, family type, and other study characteristics) affect this association?"**

<u>Comment 3</u>: Did the author address the potential overlap between samples of studies by the same author (e.g. Stocker, Tucker, Ruff, Brody)?

Author Response: We used three-level meta-analytic models to control for multiple effect sizes within the same study, but did not control for multiple publications by the same author. There were in total eight studies included in our meta-analyses that were from the same four first authors (each of those having published two studies). Including this information on (identical) authorship as a further level in the model (level 4) resulted in an estimate of the square root of this variance component of 0 (i.e., no variance was attributable to authorship). Using authorship instead of study as level 3 in the model resulted in the same estimates (to the second digit) as reported in the originally submitted manuscript. Therefore, we decided to keep the original analyses but added these additional analyses to the Results section, on page 20.

<u>Comment 4</u>: Please, deepen also theoretical implications of your results in the discussion section.

Author Response: Along with our revisions according to your first comment (major point), we tried to elaborate theoretical implications of the present meta-analysis in the discussion more comprehensively (see pages 25/26).
